# Genome-Wide Associations with Body and Fleece Weight in United States Sheep

**DOI:** 10.3390/genes16070733

**Published:** 2025-06-24

**Authors:** Gabrielle M. Becker, Daniel Schaub, J. Bret Taylor, Michelle R. Mousel, Carrie S. Wilson, Jamin A. Smitchger, Jacob W. Thorne, Brenda M. Murdoch

**Affiliations:** 1 Department of Animal, Veterinary and Food Science, University of Idaho, Moscow, ID 83844, USA; 2 US. Sheep Experiment Station, Agricultural Research Service (ARS), US. Department of Agriculture (USDA), Dubois, ID 83423, USAcarrie.wilson@usda.gov (C.S.W.); 3 Animal Disease Research Unit, Agricultural Research Service (ARS), US. Department of Agriculture (USDA), Pullman, WA 99164, USA; 4 Texas A&M AgriLife Research and Extension, San Angelo, TX 76901, USA

**Keywords:** GWAS, signatures of selection, runs of homozygosity, wool

## Abstract

**Background/Objectives**: Wool is an important product in sheep production, but the genetic mechanisms underpinning variation in wool growth are not fully understood. Identifying the genes and genomic variants that play a role in increasing fleece weight may allow for increased selection accuracy and improved economic return to producers. **Methods**: A genome-wide association study (GWAS) was conducted to investigate genetic associations with lifetime fleece weight, average fleece weight and average post-lambing ewe weight for Rambouillet, Polypay, Suffolk and Columbia ewes (*N* = 1125). Weir–Cockerham F_ST_ and runs of homozygosity (ROH) analyses were conducted to improve detection of putative wool-related signatures. **Results**: Twenty-four SNPs were identified through GWAS for lifetime fleece weight, average fleece weight and average post-lambing ewe weight. Chromosomes 2 and 6 contained ROH islands in Rambouillet, and chromosomes 2, 3 and 10 contained ROH islands in Suffolk. The F_ST_ analysis identified 18 SNPs in proximity to 37 genes of interest. **Conclusions**: Many of the SNPs and signatures of selection reported in this study are near or within current candidate genes for wool production and wool quality, including *ADAR*, *KCNN3*, *NTN1*, *SETBP1*, *TP53* and *TNFSF12*. The significant SNPs implicated by GWAS may be used to predict ewes’ potential for lifetime wool production and are suggested as candidates for further study to continue to elucidate the genetic mechanisms underlying wool production traits in United States sheep breeds.

## 1. Introduction

Shearing is an important husbandry practice that all wool-producing sheep require, regardless of their fiber quality [1]. For sheep producers operating in the Western United States (U.S.), receipts from wool sales can account for up to 13% of total revenue [2]. While wool is sold by weight, the cost of shearing is often a flat rate per animal, creating an opportunity for producers to increase their net revenue through targeting increased fleece weight selection goals. Therefore, wool production and quality should be considered when making selection decisions, especially for fine-wool breeds.

Selection towards greater fleece weights can be accomplished through selection indices that incorporate estimated breeding values and trait-associated single-nucleotide polymorphism (SNP) information. Genome-wide association study (GWAS) approaches have been used to identify associations with greasy and clean fleece weights [3,4,5,6,7], but there is a further need for studies reporting associations with lifetime wool production data. Lifetime records allow for the identification of markers or genomic regions associated with both increased average fleece weight as well as sustained lifetime production. Additionally, there is a need for further exploration of SNP associations and signatures of selection that are relevant to breeds popular in Western U.S. flocks.

To address these gaps in the current literature, lifetime records of Columbia, Polypay, Rambouillet and Suffolk ewes were utilized in GWAS models for lifetime fleece weight (LFW), average fleece weight (AFW) and average post-lambing ewe weight (PLEW) to investigate SNPs associated with direct measurements of wool weight and SNPs associated with traits that are correlated with wool weight (i.e., ewe weight). These GWAS approaches were supplemented with Weir–Cockerham F_ST_ and runs of homozygosity (ROH) analyses to identify regions potentially related to breed-specific differences in wool growth that may be detectable as recent selection signatures or as divergent allele frequencies between breeds. The results of this study contribute to the growing body of scientific literature which aims to elucidate the genetic mechanisms underpinning fiber development and wool quality.

## 2. Materials and Methods

### 2.1. Description and Analysis of Phenotypic Records

Trait records were collected from sheep managed at the USDA Range Sheep Production Efficiency Research Unit, U.S. Sheep Experiment Station (USSES) near Dubois, Idaho, USA. The USSES production records from 1999 to 2021 were used to identify Columbia (*n* = 63), Polypay (*n* = 399), Rambouillet (*n* = 481) and Suffolk (*n* = 182) ewes for analysis, for a total study sample size of *N* = 1125. All ewes were born between 1999 and 2016 and were retained within the flock for 1.5 to 9.0 years.

The LFW, AFW and PLEW were assessed from USSES records. Wool records were collected during February shearing with fleece weights taken immediately after shearing with minimal contaminants removed. Post-lambing ewe weights were measured at 30 to 40 days postpartum. In addition to the LFW, AFW and PLEW, birth year and longevity (i.e., number of years in the flock) data were carried forward to investigate potential relationships with the traits of interest. Data for LFW, AFW, PLEW, birth year and longevity were available for all 1125 ewes.

All traits were evaluated for normality using the Shapiro–Wilk test in R v4.4.1 [8]. To test for potential covariate variables, the ‘corrplot’ library was used to estimate Pearson correlation coefficients and *p*-values for longevity, LFW, AFW and PLEW traits across and within breeds [9], and ‘oneway.test’, ‘aov’ and ‘TukeyHSD’ were used for ANOVA and Tukey HSD testing of LFW, AFW and PLEW mean differences between breeds and birth years.

### 2.2. Genotype Data Preparation for Analyses

#### 2.2.1. Genotype Preparation for Genome-Wide Association Study

The sample collection, DNA extraction and genotyping methods for the data utilized in this study have been previously described [10,11]. In brief, genotyping was conducted by Geneseek Inc. (Lincoln, NE, USA) with the OvineHD BeadChip (Illumina Inc., San Diego, CA, USA) containing 606,006 SNP markers. Genotype data were filtered with plink v2 [12] to remove markers with a minor allele frequency (MAF) less than 1% (‘--maf’) and/or a call rate (CR) less than 90% (‘--geno’). All samples had a CR greater than 90%. Following quality control, 501,560 autosomal SNPs were carried forward for GWAS. To confirm sample breed identity and assess population structure, a principal component analysis (PCA) was conducted in plink (‘--pca’) and visualized in R with ‘ggplot’ [13]. The PCA is provided as Supplementary Figure S1.

#### 2.2.2. Genotype Preparation for Signature of Selection Analyses

To improve the breed representation for signatures of selection analyses, the OvineHD BeadChip data of 302 Suffolk samples were retrieved from publicly available data to supplement the USSES data [14]. Due to the limitations of their sample size and the lack of publicly available breed data, the Columbia samples were not included in the ROH or F_ST_ analyses. For both the ROH and F_ST_ analyses, the USSES/Repository data were filtered to remove markers or samples with CR less than 90%, and for F_ST_ only, markers were filtered to remove those with MAF less than 1%. The ROH dataset did not exclude low-MAF markers as this may interfere with ROH detection [15,16]. The ROH analysis included 496,238 SNPs, the F_ST_ analysis included 489,470 SNPs and both analyses included 1364 sheep, comprising 399 Polypay, 481 Rambouillet and 484 Suffolk sheep.

### 2.3. Genome-Wide Association Studies for Ewe Fleece and Body Weight

The GWASs for LFW, AFW and PLEW were conducted in GCTA v1.94.1 [17] with the mixed linear model association (MLMA) analysis implemented with the leaving-one-chromosome-out (LOCO) genomic relationship matrix approach (‘--mlma-loco’) to address population stratification. To account for significant relationships within the data, the GWASs for LFW and AFW included birth year, longevity and PLEW as covariates while the GWAS for PLEW included birth year and longevity. To evaluate GWAS results for pleiotropic signals for ewe weight and fleece weights, the LFW and AFW GWASs were conducted in a second iteration without PLEW used as a covariate. All other parameters were the same between analyses.

Genome-wide significance (*p*-values < 9.969 × 10^−8^) was determined by the Bonferroni *p*-value correction, given by alpha (0.05) divided by the number of SNPs (501,560 SNPs). Similarly, chromosome-wide significance (*p*-values < 8.867 × 10^−7^) was determined by the Bonferroni *p*-value correction based on the number of SNPs on the largest chromosome (alpha 0.05/SNP count 56,387). The GWAS results were visualized in R with the package ‘CMplot’ [18]. Linkage disequilibrium (LD) and haplotype frequencies for significant GWAS SNPs were evaluated using the *r^2^* statistic calculated through plink with (‘--ld’). Descriptive statistics of trait averages and standard deviations were calculated for each breed genotype group using ‘dplyr’ and ‘purrr’ in R for SNPs of interest [19,20] (Supplementary File S3).

### 2.4. Runs of Homozygosity Analysis

The ROH analysis was conducted at two levels, with both analyses using the sliding window method in the ‘detectRuns’ package in R version 4.4.1 [21]. The first and most conservative analysis required each ROH to achieve a minimum length of 1,000,000 bp and is identified by ‘ROH (1 Mb)’. Additional parameters included a sliding window size of 50 SNPs, a minimum of 60 SNPs within an ROH, an overlapping windows threshold of 0.05, a minimum density of 1 SNP per 50 kbp and a maximum of two opposite and one missing SNPs per window. The second analysis required a minimum length of 500,000 bp and is identified by ‘ROH (0.5 Mb)’. The ROH (0.5 Mb) analysis otherwise used the same parameters as ROH (1 Mb), with the following exceptions: a sliding window size of 40 SNPs and a minimum of 50 SNPs per ROH. Various thresholds have been used to define ROH signatures in previous studies, including the top 1% of SNPs or SNPs with greater than 30% or 50% ROH incidence per breed [22,23,24,25]. In order to limit results to highly conserved signals, the current study required an ROH to be present in at least 70% of the breed to be considered as an ROH island. To assist with interpretation of ROH results, the expected homozygosity, observed homozygosity and the method-of-moments inbreeding coefficient (F_MOM_) were estimated in plink (‘--het’), and ROH-based inbreeding coefficient (F_ROH_) was estimated from the ROH calculations in detectRuns from the ROH (1 Mb) parameters. Statistical analyses were conducted using *t*-test, ANOVA and Tukey HSD in R to analyze differences between breeds and between inbreeding coefficient estimations. Inbreeding distributions were visualized with ggplot2, and the ROH results were visualized as Manhattan plots with CMplot in R.

### 2.5. Weir–Cockerham F_ST_ Analysis

Weir–Cockerham F_ST_ analysis was conducted in plink with (--fst method=wc report-variants’) and (‘--pheno’) parameters. Negative F_ST_ estimates were considered to be zero. Within each pairwise breed analysis, an SNP was identified as an outlier when the F_ST_ estimate was greater than four standard deviations above the mean and the genotypes were found to vary significantly between breeds by Fisher’s Exact Test by the Bonferroni-adjusted threshold (*p*-value < 1.02 × 10^−7^). Statistical testing was conducted with ‘fisher.test’ in R. In order to limit F_ST_ interpretation as much as possible to wool-related breed differences, the significant Rambouillet–Suffolk, significant Rambouillet–Polypay and insignificant Suffolk–Polypay pairwise comparisons were compared. To be carried forward for further interpretation, an SNP was required to be significant in both the Rambouillet–Suffolk and Rambouillet–Polypay analyses by *p*-value and F_ST_ statistic and insignificant by F_ST_ statistic within the Suffolk–Polypay analysis. Rambouillet are a fine-wool breed, while both Polypay and Suffolk produce a medium wool (Table 1) [2,26,27,28]. The significant/insignificant analysis design was intended to identify SNPs with allele frequencies related to wool status differences between the breeds and avoid or minimize carrying forward other differences.

### 2.6. Genomic Context of Significant Results

The reference genome ARS-UI_Ramb_v3.0 was used to investigate the genomic context of study results [30]. Significant SNPs from GWAS, ROH and F_ST_ analyses were examined with the same methods. For each significant SNP, the genomic region immediately ±50,000 bp was defined as the region of interest, and any genes overlapping with or contained within the region were carried forward. Genes implicated by GWAS, ROH or F_ST_ analyses were compared against the previous literature to evaluate the level of support currently existing for these genes as candidates for wool quality, follicle growth or related traits in sheep or other species.

## 3. Results

### 3.1. Statistical Analyses of Phenotypic Records

**Figure 1:**
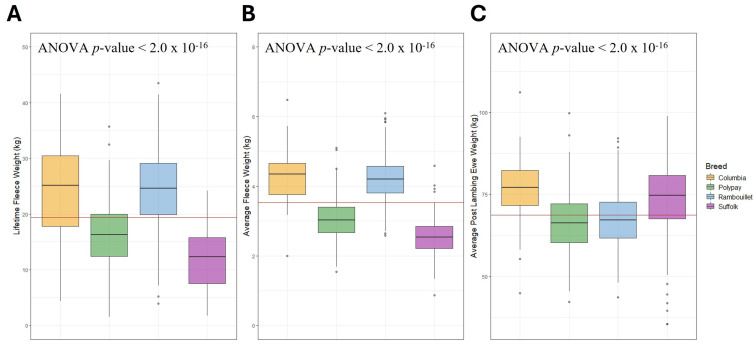
Distribution of LFW, AFW and PLEW in kilograms by breed. The across-breed average for each trait is represented by the horizontal red line. (**A**) Distribution of LFW. (**B**) Distribution of AFW. (**C**) Distribution of PLEW.

The distribution of ewe data for LFW, AFW and PLEW varied within and between breeds (Table 2). By descriptive statistics, Rambouillet ewes had the greatest average LFW (24.20 ± 6.92 kg), and Columbia ewes had the greatest average AFW (4.26 ± 0.72 kg) as well as the greatest average PLEW (76.60 ± 9.16 kg). The ANOVA models for LFW, AFW and PLEW by ewe breed were significant with *p*-values < 2 × 10^−16^ (Figure 1). In post hoc Tukey HSD testing, all breeds differed significantly for LFW and AFW except for the Rambouillet and Columbia ewes, who were not significantly different (LFW *p*-value = 0.995, AFW *p*-value = 0.846). The *p*-values of all other breed comparisons were similar and varied from 9.66 × 10^−13^ to 3.75 × 10^−13^ for LFW and from 4.13 × 10^−13^ to 3.75 × 10^−13^ for AFW (Supplementary Table S1). For PLEW, all pairwise breed comparisons were significant except the Suffolk–Columbia (*p*-value = 0.062) and the Rambouillet–Polypay (*p*-value = 0.175) analyses. The most significant difference was identified between the Suffolk and Polypay ewes (*p*-value = 4.19 × 10^−13^), followed by the Polypay and Columbia (*p*-value = 4.26 × 10^−13^). The ANOVA analyses with birth year also yielded significant associations with LFW (*p*-value < 2.2 × 10^−16^), AFW (*p*-value = 9.62 × 10^−16^) and PLEW (*p*-value = 2.2 × 10^−16^).

**Figure 2:**
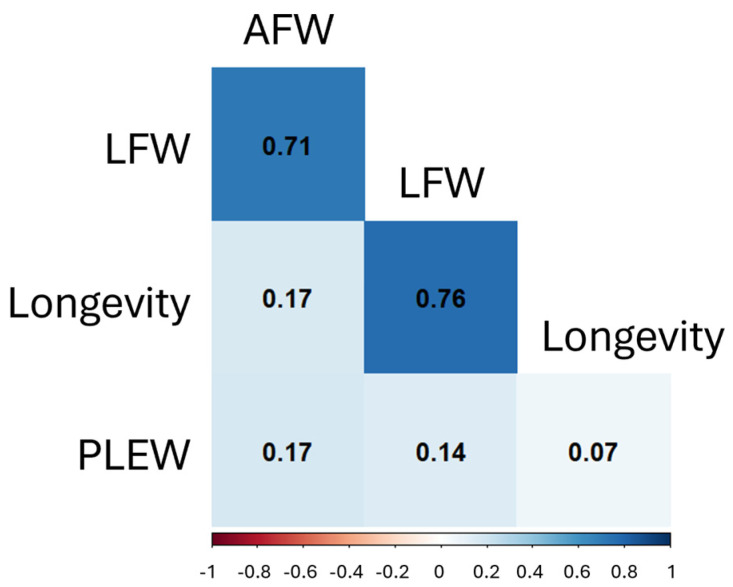
Pearson correlation coefficients between AFW, LFW, PLEW and longevity across breeds. All correlations were significant with *p*-values < 0.05.

Correlation testing was conducted for LFW, AFW, PLEW and longevity. All correlations were significant (*p*-values < 0.05) in both the within-breed and across-breed analyses (Figure 2). In the across-breed data, LFW and longevity had the strongest correlation (*r* = 0.76), followed by LFW and AFW (*r* = 0.71), and PLEW with longevity had the weakest correlation (*r* = 0.07). The LFW to AFW correlations were of similar strength in each breed (*r* = 0.46 to *r* = 0.54), while correlations of LFW or AFW to PLEW were more variable (*r* = 0.09 to *r* = 0.57 and *r* = 0.21 to *r* = 0.43, respectively). All correlation coefficients are provided in Supplementary Figure S2, and *p*-values are provided in Supplementary Table S2.

### 3.2. Genome-Wide Association Studies for Ewe Fleece and Body Weight

**Figure 3:**
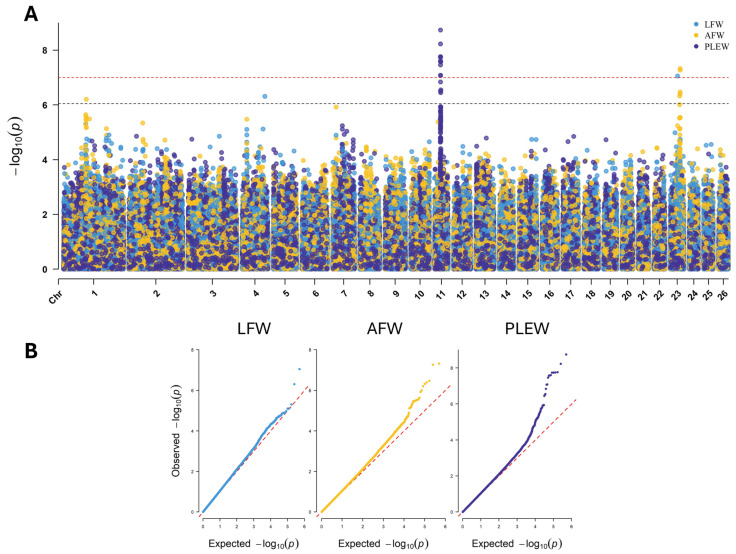
Results of GWAS for LFW, AFW and PLEW. (**A**) Manhattan plot displaying the SNP *p*-values against their chromosomal position for the LFW (light blue), AFW (yellow) and PLEW (dark blue) models. The genome-wide significance threshold is indicated by the horizontal red line (*p* < 9.97 × 10^−8^), and the chromosome-wide significance threshold is indicated by the black horizontal line (*p* < 8.87 × 10^−7^). (**B**) Q-Q plot displaying the expected *p*-value against the observed *p*-value for the LFW, AFW and PLEW models.

Through the GWAS for LFW, AFW and PLEW, 15 genome-wide significant SNPs and 9 chromosome-wide significant SNPs were identified on chromosomes 1, 4, 11 and 23 (Figure 3A). The QQ plot for each GWAS showed appropriate control of *p*-value inflation (Figure 3B). The largest significant region was identified in PLEW GWAS and comprised 13 SNPs from 27,131,851–27,269,772 bp chromosome 11 (Table 3). Significant SNPs within this region are positioned within exons of the genes *SOX15*, *EIF4A1*, *CD68* and *MPDUI*; within introns of *CD68*, *FXR2*, *TP53*, *WRAP53* and *DNAH2*; and in the 3′ region of the gene *SHBG* (Table 4). The effect allele was present at a frequency of 0.39 to 0.49, the effect size (beta) ranged from −2.65 to 2.15 kg and the *p*-values ranged from 3.16 × 10^−7^ to 1.83 × 10^−9^ for these SNPs. Beyond this region, the PLEW GWAS identified three additional SNPs located on chromosome 11. These SNPs are positioned exonic of *MFSD6L* (rs161050016), intronic of *NTN1* (rs420045693) and exonic of *GSGIL2* (rs420358530). Analysis of LD between GWAS SNPs on chromosome 11 identified substantial LD between markers in the 27.13–27.27 Mb region (*r^2^* = 0.39–1.0) (Supplementary File).

The LFW GWAS identified two significant SNPs on chromosomes 4 and 23 (Table 3). The SNP rs423559749, an exonic variant within *LOC114114557* (*TRBV6-2*), had an allele frequency of 0.03, effect size of −2.22 kg and *p*-value of 4.87 × 10^−7^. The SNP rs401427029, an intronic variant of *TTC39C*, had an allele frequency of 0.41, effect size of 0.87 kg and *p*-value 8.86 × 10^−8^. Six SNPs were identified through AFW GWAS, with four SNPs contained within close proximity on chromosome 23 (44,917,577–44,977,724 bp). These four SNPs are intronic of *SETBP1*, were present at frequencies of 0.27 to 0.34 and had effect sizes of −0.15 to −0.16 kg and *p*-values of 3.99 × 10^−7^ to 4.73 × 10^−8^. The remaining significant SNPs were positioned 5′ of *ADAR* on chromosome 1 and intronic of *PIEZO2* on chromosome 23 (Table 4).

The results of LFW and AFW GWASs were similar when analyzed without PLEW as a covariate (Supplementary File S3). For LFW, the SNP rs401427029 reached chromosome-wide significance instead of genome-wide significance, and rs423559749 was not significant. No additional signals were detected. For AFW, the SNP rs430733414 achieved genome-wide significance rather than chromosome-wide significance, and rs421331549 and rs407948628 were identified as chromosome-wide significant SNPs. While rs421331549 was not identified in the initial model, it is positioned 9796 bp from rs407174590, which was identified in the initial AFW model. Both of these SNPs are located in proximity to *ADAR*, with rs421331549 being intronic and rs407174590 being positioned 5′ of the gene. Similarly, rs407948628 was not identified in the initial model but is positioned 4296 bp from rs418816169, which was identified in both models. These SNPs are both positioned within an intron of *PIEZO2*. While there were some differences in the individual SNP associations between these GWAS approaches, the genomic regions containing significant genes were the same in each approach.

### 3.3. Runs of Homozygosity Analysis

**Figure 4:**
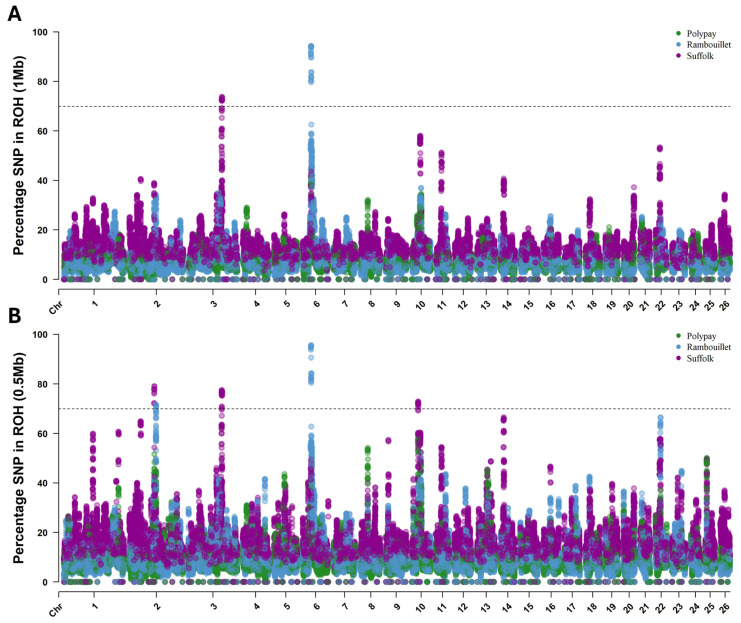
Results of ROH for Rambouillet, Suffolk and Polypay sheep. The threshold for calling an ROH island (70%) is represented by the dotted line in each panel. (**A**) Results for the ROH (1 Mb) approach. (**B**) Results of the ROH (0.5 Mb) approach.

An SNP was determined to be within an ROH island if it belonged to an ROH in 70% or more of the individuals of the breed by either ROH (1.0 Mb) (Figure 4A) or ROH (0.5 Mb) (Figure 4B) parameters. In total, the ROH analyses identified five ROH islands in the Rambouillet and Suffolk breeds, with two islands identified by ROH (1.0 Mb) and an additional three islands identified by ROH (0.5 Mb).

The largest and most highly conserved ROH island was identified within the Rambouillet (Table 5). This signature was 1.64 Mb in length and comprised 199 SNPs on chromosome 6. While the SNP composition and size of this ROH island were consistent between ROH analysis levels, the percentage of individuals included was slightly variable. By ROH (1.0 Mb), SNPs within the signature were called in an ROH in 79.63% to 94.39% of the breed, while SNPs were called within an ROH in 80.46% to 95.63% of the breed by ROH (0.5 Mb) parameters (Table 5).

In Suffolk, the most highly conserved island by ROH (1.0 Mb) parameters was identified on chromosome 3. This signature comprised 141 SNPs (153,959,687–154,922,651 bp, 0.96 Mb length) and was present in 72.11% to 73.76% of the breed. By the ROH (0.5 Mb) parameters, this island included 157 SNPs (153,875,227–154,922,651 bp, 1.05 Mb length) and was present in up to 77.48% of the breed.

The most highly conserved ROH island identified by ROH (0.5 Mb) parameters comprised 98 SNPs on chromosome 2 (114,908,538–115,694,442 bp, 0.79 Mb length) and was present within 72.11% to 79.13% of Suffolk. Two additional ROH islands were identified by ROH (0.5 Mb) parameters: on chromosome 2 (123,322,344–123,534,483 bp) in up to 71.73% of Rambouillet and on chromosome 10 (29,230,492–29,662,445 bp) in up to 72.93% of Suffolk (Table 5). Of note, the ROH islands identified on chromosome 2 in Rambouillet and Suffolk samples did not share any overlap between breeds: the beginning of the ROH island in Rambouillet was positioned 7.63 Mb past the end of the ROH island in Suffolk. While there was no overlap based on the ROH island threshold of 70%, there were some commonalities below this threshold. Specifically, up to 42.0% of Rambouillet samples had ROH called for SNPs within the ROH island identified in Suffolk, and up to 30.17% of Suffolk samples had ROH called within the ROH island signature of Rambouillet. Additionally, over 41% of Polypay and nearly 50% of Suffolk samples had ROH called within the Rambouillet ROH island on chromosome 6, and over 57% of Polypay samples had ROH called within the Suffolk ROH island on chromosome 10.

The five ROH islands identified through ROH (1.0 Mb) and ROH (0.5 Mb) analyses contained 32 genes (Table 6). Genes identified through ROH analysis included the following: *LOC106990902* (*MSX2*) and *LOC101122056* (*TMED2*) on chromosome 2; *HMGA2*, *MSRB3* and *WIF1* on chromosome 3; *LAP3*, *LCORL*, *NCAPG* and *MED28* on chromosome 6; and *RXFP2* on chromosome 10.

As ROH can be influenced by inbreeding, the F_MOM_ and F_ROH_ inbreeding coefficients were estimated to evaluate the degree of homozygosity among the Polypay, Rambouillet and Suffolk samples analyzed. Observed homozygosity was the greatest in Suffolk (69.97% ± 1.20) and similar between Polypay (67.38 ± 0.77) and Rambouillet (67.67 ± 0.70) (Supplementary Table S3). The Suffolk were found to have the greatest average inbreeding coefficients by both F_MOM_ (0.107) and F_ROH_ (0.116). Similarly to previous reports, the F_MOM_ estimations were significantly lower than the inbreeding coefficients estimated by the F_ROH_ approach [25], with a *t*-test *p*-value < 2.2 × 10^−16^ and a mean inbreeding of 0.060 in F_MOM_ and 0.086 in F_ROH_ (Supplementary Figure S3). Inbreeding estimations between breeds varied significantly in both F_MOM_ (ANOVA *p*-value < 2.0 × 10^−16^) and F_ROH_ (ANOVA *p*-value < 2.0 × 10^−16^). All breed comparisons were significantly different (Supplementary Table S4), with the exception of the F_ROH_ Rambouillet–Polypay comparison (Tukey HSD *p*-value = 0.93). Overall, the homozygosity analysis indicated that the degree of inbreeding in the study samples was consistent with the published estimates for these breeds [25,31]. Therefore, the detection of long ROH within the Rambouillet and Suffolk breeds was not due to a high degree of inbreeding.

### 3.4. Weir–Cockerham F_ST_ Analysis

F_ST_ analysis was conducted from the results of Rambouillet–Suffolk, Rambouillet–Polypay and Suffolk–Polypay pairwise analyses. Through this approach, 18 SNPs with *p*-values < 2.2 × 10^−16^ and F_ST_ estimates at least four standard deviations above the mean in Rambouillet–Suffolk (F_ST_ > 0.470) and Rambouillet–Polypay (F_ST_ > 0.325), as well as F_ST_ less than four standard deviations above the mean in Suffolk–Polypay (F_ST_ < 0.450), were identified (Table 7). Fifteen of these SNPs are positioned within ± 50,000 bp of a gene, and six are positioned within a gene, including the following: exonic of *CCND2*, intronic of *TMEM178B*, intronic of *PEPD* (two SNPs), intronic of *PDE4D* and intronic of *TRIM38*.

The genotypic frequencies for each SNP were evaluated to identify patterns between breeds. The Rambouillet showed the greatest within-breed similarity, with genotypic frequencies for the most common genotype ranging from 0.593 to 0.985 and averaging 0.835 (Supplementary File). Additionally, the most common genotype for each SNP in Rambouillet was homozygous. Conversely, the Polypay and Suffolk showed less similarity within their breeds: the genotypic frequencies for the most common genotypes ranged from 0.454 to 0.561 (average 0.512) in Polypay and 0.462 to 0.654 (average 0.525) in Suffolk. The Polypay were predominantly heterozygous at 17 of the 18 SNPs, while the Suffolk were predominantly heterozygous at 13 of the 18 SNPs. At SNP rs159911297 (exonic of *CCND2*), the Rambouillet had AA genotypes (0.821), while the Polypay and Suffolk had predominantly AG genotypes (0.561 and 0.489). Similar trends were observed for SNPs rs401322014 (intronic of *PEPD*), rs429950267 (intronic of *PEPD*), rs417106434 (intronic of *PDE4D*) and rs413553624 (intronic of *TRIM38*), with the Rambouillet being homozygous (0.684 to 0.904) and the Polypay and Suffolk being heterozygous (0.454 to 0.546 and 0.462 to 0.511, respectively). At the SNP rs408105992 (intronic of *TMEM178B*), Polypay had largely AG and GG genotypes (0.491 and 0.378), Rambouillet had mostly AA and AG (0.717 and 0.264) and Suffolk were mostly GG and AG (0.577 and 0.396). Overall, the significant F_ST_ estimates and dramatic differences in genotypic frequencies at these SNPs suggest the potential for these variants or nearby genomic features to contribute to Rambouillet’s capacity for greater fleece weights as compared to Polypay and Suffolk. Further work is necessary to conduct functional validation of the relationship between these F_ST_ signals and wool growth traits in sheep.

## 4. Discussion

Wool production is a complex biological process that can be affected by many factors, including genetics, nutrition, health status, physiological stage and nutrition during fetal development [1,32]. The aim of the current study was to use SNP data and lifetime ewe production records to improve understanding of the genetic mechanisms contributing to variations in wool growth. Overall, this study reports 24 genome-wide and chromosome-wide significant SNPs associated with LFW, AFW and PLEW through GWAS, five ROH islands with high conservation in Rambouillet and Suffolk breeds and 18 SNPs achieving stringent outlier and significance thresholds in F_ST_ analyses. These three approaches identified 118 genes of interest for their association with wool weight, body weight or signatures of selection between medium- and fine-wool sheep.

### 4.1. Genes of Interest Identified by GWAS for LFW, AFW and PLEW

Body size and wool production have been shown to be correlated, with larger adults producing heavier fleeces with greater fiber diameter, staple strength and staple length [33]. Additionally, selection for sheep with finer wool tends to come with the consequence of reduced fleece and body weights [34]. While the strength of correlation of PLEW with AFW and LFW varied between breeds, there were significant relationships between these traits for both the within-breed and across-breed analyses. Therefore, the GWAS approach of this study utilized the fleece weight traits LFW and AFW to investigate associations with greater average and sustained greasy fleece production, and PLEW was investigated due to the relationship between body size and fleece weight. The GWAS conducted with these data identified 24 significant SNPs and suggested 50 potential genes of interest.

While the fleece weight traits LFW and AFW were significantly correlated, they detected unique SNP associations through GWAS. Of note, both analyses identified variants within different regions of chromosome 23: in LFW, SNP rs401427029 at 32.98 Mb, and in AFW, SNP rs418816169 at 42.86 Mb and SNPs rs430733414, rs403221986, rs427745519 and rs413519109 at 44.92–44.98 Mb. These results suggest the benefit of investigating fleece weights through multiple approaches due to the opportunity to detect unique signatures even between correlated traits.

The genome-wide significant SNPs identified through LFW and AFW showed different trends in the direction of their effect sizes. The SNP rs401427029 in LFW had a positive effect of 0.87 kg associated with the C allele, which was consistent across all breeds: within each breed group, the mean LFW for sheep with the CC genotype was greater than the mean LFW for sheep with AC or AA genotypes (Supplementary File). In the AFW results, the SNPs rs403221986 and rs427745519 had effect sizes of −0.16 associated with the T alleles. At SNP rs403221986, the average AFW was greatest for ewes with the CC genotype, with the exception of a single Columbia ewe that had a TT genotype (5.53 kg). At SNP rs427745519, the negative effect of the T allele was driven by Rambouillet (CC, 4.23 kg; CT, 4.11 kg; TT, 3.41 kg) and Polypay (CC, 3.21 kg; CT, 3.01 kg; TT, 2.93 kg). Within Suffolk, ewes with one or more copies of the T allele had the greatest average AFW (CC, 2.47 kg; CT, 2.58 kg; TT, 2.54 kg), and Columbia ewes had no allele-associated pattern (CC, 4.35 kg; CT, 4.07 kg; TT, 4.39 kg), which may have been influenced by their comparatively smaller representation within the study. The different effect directions within versus between breeds illustrate the importance of conducting research across diverse breed groups, as SNPs may have different relationships with the trait of interest depending on the breed background.

Many of the genes implicated by GWAS have been previously suggested through signature of selection or differential gene expression (DEG) studies for wool growth and related traits in sheep and other ruminant species (Table 8). The genes with the greatest number of previous reports included potassium calcium-activated channel subfamily N member 3 (*KCNN3*) identified in the AFW GWAS, and netrin 1 (*NTN1*) and TNF superfamily member 12 (*TNFSF12*) both identified in the PLEW GWAS. In human patients, *KCNN3* and *NTN1* have both been implicated in conditions related to hair growth, including onychodystrophy syndromes and deafness. Variants within *KCNN3* have been associated with Zimmermann–Laband syndrome, which can be characterized by many phenotypes, including hypertrichosis [35], and *NTN1* has roles in cochlear hair cell survival [36]. Additionally, these genes have been connected with body size and muscling traits in sheep. The gene *NTN1* has been associated with post-weaning gain [28], both *NTN1* and *TNFSF12* have been associated with sheep body size [37,38] and *KCNN3* has been identified for expression in the *semimembranosus* and *longissimus dorsi* muscle tissue of Callipyge lambs [39].

The most significant SNPs identified by LFW, AFW and PLEW were rs401427029, rs427745519 and rs162128226, respectively. The SNP rs401427029 is an intronic variant of *TTC39C*, a gene which has been previously identified as a candidate for growth traits in Chubao black-head goats [48]. The SNP rs427745519 is positioned within an intron of *SETBP1*, which encodes the transcription factor SET binding protein 1. Of note, *SETBP1* was also a gene of interest by F_ST_ analysis. Variants within the 3′ region of *SETBP1* have been associated with early-onset androgenetic alopecia in human patients [49]. Several other inherited and somatic diseases have been linked to *SETBP1* variants, including *SETBP1* haploinsufficiency disorder, Schinzel Giedion Syndrome and some hematological malignancies and cancers in adults [50], and *SETBP1* was reported for expression in Yak hair follicles collected at different phases of development [42]. Finally, the SNP rs162128226 is an intronic variant of *TP53*. Work in mice models has characterized TP53-dependent cell death occurring in proliferative keratinocytes [51], and expression of *TP53* has been reported in Suzhou sheep skin [44]. The association of these SNPs with LFW, AFW and PLEW in the current study suggests the potential for these SNPs to be involved in biological pathways relevant to production traits in sheep. Further work is needed to fully understand the consequence of the SNPs identified in these regions.

The SNP rs423559749, which achieved chromosome-wide significance for LFW, is an exonic variant of *LOC114114557* (*TRBV6-2*). The region ± 50,000 bp of rs423559749 contains 12 other T-cell receptor beta variable-like (*TRBV*-like) genes, of which *LOC101114438* (*TRBV7-9*) has been previously reported in a signature of selection analysis for wool fineness [43]. Changes in the expression of *TRBV5-7* and *TRBV2* have been noted in human patients with early-stage to advanced-stage mycosis fungoides, a type of cutaneous T-cell lymphoma that mainly affects the hair follicle [52,53]. While the importance of *TRBV*-like genes to wool growth is not defined, the anagen hair bulb is considered an immune privileged site [54], and previous studies have identified other immune genes to be differentially expressed between wooly and woolless skin samples of sheep [55]. The association of this *TRBV* gene-rich region is intriguing given the importance of immune regulation to hair follicle cycling and hair follicle stem cell activity [56].

### 4.2. Signatures of Selection

Signatures of selection analyses can be used to identify genomic regions that have experienced recent selection pressure through the presence of consecutive segments of increased homozygosity (ROH) or identify signatures of allelic frequency differences between groups (F_ST_) [57,58]. These analyses were used to identify selection signatures related to differences between the medium- (Polypay, Suffolk) and fine-wool (Rambouillet) breeds of this study.

The ROH region identified on chromosome 6 in Rambouillet has been previously reported as a signature of selection in sheep and cattle [25,59,60,61,62,63]. Genes underlying this region have been associated with many production traits, including growth and body weight, milk solids percentage, somatic cell score and meat productivity [64,65,66,67,68,69]. Four genes from this region have been associated with wool production through transcriptomics or association studies: leucine aminopeptidase 3 (*LAP3*), ligand-dependent nuclear receptor corepressor-like (*LCORL*) protein, mediator complex subunit 28 (*MED28*) and non-SMC condensin I complex subunit G (*NCAPG*) [40,41,42,44,55]. Additionally, *LAP3* and *MED28* have been associated with live weight in Merino sheep [70]. While this region has strong associations with multiple production traits, the associations with body size and presence of genes with detectable expression in skin suggest that this ROH island may also be involved in recent or ongoing selection towards wool-related traits highly conserved in Rambouillet sheep.

Genes within Suffolk ROH islands with previous reports in wool-related transcriptomics, signatures of selection and association studies include high-mobility group AT-hook 2 (*HMGA2*), LEM domain-containing protein 3 (*LEMD3*), LOC101110773 (elongation factor 1-alpha 1; *EEF1A1*), LOC106990902 (homeobox protein MSX-2-like; *MSX2*), methionine sulfoxide reductase B3 (*MSRB3*), relaxin family peptide receptor 2 (*RXFP2*) and WNT inhibitory factor 1 (*WIF1*) [40,41,42,43,44,45,71,72,73]. While it is possible that these ROH islands are associated with the lower fleece weights observed in the Suffolk ewes, it is also likely that these signatures are driven by unrelated, breed-specific traits that are under stronger selection in Suffolk than wool weight. Future work will be helpful to illuminate the potential traits and direction of selection implicated by these signatures.

Many of the genes implicated by F_ST_ analysis have been previously reported by transcriptomics studies of genes associated with wool and fiber growth in ruminant species. Specifically, *ABCG2*, *CCND2*, *GABRP*, *KCNIP1*, *PEPD*, *PPM1K* and *SETBP1* are reported as DEG in Yak hair follicles [42], *ABCG2*, *CCND2*, *ETFA*, *KCNIP1*, *MBNL2*, *PDE4D*, *PEPD*, *PPM1K* and *SCAPER* were expressed in Hu sheep skin samples [44], *H1-1*, *KCNIP1* and *SCAPER* were differentially expressed in a study of Pashima fiber initiation [45], and finally, *BCLAF1*, *CCND2*, *ETFA*, *ISL2*, *KCNIP1*, *PDE4D*, *PEPD*, *PPM1K*, *SCAPER* and *TIGAR* were reported in a transcriptomics study of multiple species [41]. The identification of outlier F_ST_ signals within or near these genes, together with previous transcriptomics reports, suggests the importance of these regions for wool production in sheep. The allelic and genotypic frequency differences between Rambouillet, Polypay and Suffolk in these regions may have phenotypic consequences, although further work is needed to discover the specific relationships between F_ST_ SNPs and wool growth parameters in these breeds.

## 5. Conclusions

Selection for average and lifetime fleece weights can improve profitability for producers of fine-wool sheep. Understanding the SNPs and genes related to variation in wool growth would improve understanding of this important biological product and facilitate the creation of more accurate indices to balance fleece weight gains with other production goals. This study used GWAS to identify genetic variants potentially associated with relevant traits, including lifetime and average greasy fleece weight and average post-lambing ewe weight. Additionally, this study incorporated ROH and F_ST_ signatures of selection analyses to identify genomic regions with homozygosity or allele frequency differences between medium- and fine-wool breeds. Many of the genes proposed by these analyses have been previously reported in transcriptomics studies relevant to hair follicle growth or have been implicated by previous signatures of selection and association studies for related traits. This study provides further evidence towards the importance of these reported candidate genes and suggests SNPs of interest for further consideration.

## Figures and Tables

**Table 1:** Characteristics and origin of sheep breeds in this study. FD, fiber diameter; GF Wt, greasy fleece weight; SL, staple length. Breed characteristics are cited from the American Sheep Industry Association (sheepusa.org) and [2,26,27,29].

Breed	Class	Wool Type	AVG FD (µm)	GF Wt (kg)	Yield (%)	SL (cm)	Background and Origin
Columbia	Dual-purpose	Medium	30–23	5.4–7.3	45–55	10.2–15.2	Established in the U.S. from Lincoln × Rambouillet crosses around 1912.
Polypay	Dual-purpose	Medium (variable)	33–22	2.7–4.5	57	7.6–12.7	Established in the U.S. from Targhee × Dorest and Rambouillet × Finnsheep crosses around 1968.
Rambouillet	Fine-wool, dual-purpose	Fine	24–19	4.5–6.8	45–55	6.4–10.2	Originated in France and Germany from the Spanish Merino. The U.S. population was established around 1840.
Suffolk	Meat, dual-purpose	Medium	33–26	1.8–3.6	50–60	5.0–8.9	Originated in England from Southdown × Norfolk crosses. The U.S. population was established around 1888.

**Table 2:** Descriptive statistics of GWAS traits calculated across-breed and within-breed.

		LFW (kg)	AFW (kg)	PLEW (kg)
All	Average ± SD	19.36 ± 8.01	3.53 ± 0.89	68.66 ± 9.64
	Min	1.54	0.86	35.38
	Max	43.54	6.49	106.14
Rambouillet	Average ± SD	24.20 ± 6.92	4.20 ± 0.61	67.71 ± 8.44
	Min	3.90	2.59	43.54
	Max	43.54	6.10	92.08
Columbia	Average ± SD	23.99 ± 8.98	4.26 ± 0.72	76.60 ± 9.16
	Min	4.35	2.00	44.91
	Max	41.55	6.49	106.14
Polypay	Average ± SD	16.19 ± 5.60	3.05 ± 0.56	66.45 ± 9.16
	Min	1.54	1.54	42.18
	Max	35.74	5.11	99.79
Suffolk	Average ± SD	11.90 ± 5.21	2.55 ± 0.52	73.27 ± 10.87
	Min	1.72	0.86	35.38
	Max	24.13	4.58	98.88

**Table 3:** Results from LFW, AFW and PLEW GWASs. Results indicated by (^) are significant by the chromosome-wide threshold, and all other results are significant by the genome-wide threshold. A1, effect allele; A2, the other allele; Freq, the frequency of A1; Beta, the SNP effect; SE, standard error estimate.

Trait	SNP	Chr	bp	A1	A2	Freq	Beta	SE	*p*-Value
LFW	rs423559749	4	106,967,335	A	G	0.03	−2.22	0.44	4.87 × 10^−7^ ^
	rs401427029	23	32,984,219	C	A	0.41	0.87	0.16	8.86 × 10^−8^
AFW	rs407174590	1	104,876,990	C	T	0.34	0.13	0.03	6.27 × 10^−7^ ^
	rs418816169	23	42,855,873	C	T	0.27	−0.16	0.03	4.73 × 10^−7^ ^
	rs430733414	23	44,917,577	A	G	0.33	−0.15	0.03	3.99 × 10^−7^ ^
	rs403221986	23	44,936,182	T	C	0.28	−0.16	0.03	5.39 × 10^−8^
	rs427745519	23	44,938,238	T	C	0.34	−0.16	0.03	4.73 × 10^−8^
	rs413519109	23	44,977,724	C	A	0.27	−0.15	0.03	3.37 × 10^−7^ ^
PLEW	rs161045311	11	27,131,851	T	A	0.48	−2.45	0.44	2.48 × 10^−8^
	rs430590929	11	27,132,389	C	T	0.48	−2.42	0.44	3.45 × 10^−8^
	rs161045330	11	27,132,449	T	C	0.48	−2.48	0.44	1.82 × 10^−8^
	rs161045389	11	27,135,432	C	A	0.49	−2.45	0.43	1.73 × 10^−8^
	rs401081841	11	27,150,195	A	G	0.48	−2.48	0.44	1.82 × 10^−8^
	rs419921875	11	27,153,661	G	A	0.48	−2.48	0.44	1.78 × 10^−8^
	rs421850429	11	27,175,522	T	C	0.48	−2.45	0.44	2.64 × 10^−8^
	rs162128226	11	27,199,959	C	T	0.40	−2.65	0.44	1.83 × 10^−9^
	rs417779412	11	27,201,632	A	C	0.39	−2.59	0.44	5.88 × 10^−9^
	rs399877817	11	27,215,959	A	G	0.47	−2.21	0.43	3.16 × 10^−7^ ^
	rs161046190	11	27,217,384	C	T	0.49	−2.27	0.43	1.46 × 10^−7^ ^
	rs426045550	11	27,262,177	A	G	0.43	2.33	0.44	8.26 × 10^−8^
	rs425193551	11	27,269,772	T	C	0.43	2.33	0.44	8.47 × 10^−8^
	rs161050016	11	28,113,457	G	C	0.47	−2.18	0.43	3.57 × 10^−7^ ^
	rs420045693	11	28,383,822	C	T	0.39	−2.34	0.42	2.66 × 10^−8^
	rs420358530	11	28,784,756	A	G	0.45	2.15	0.42	2.82 × 10^−7^ ^

**Table 4:** Genomic context of SNPs identified by GWAS for LFW, AFW and PLEW. Individual SNPs within ± 50,000 bp of each other were considered to belong to the same genomic region. For each region, any genomic elements present within 50,000 bp of the flanking SNPs were identified for consideration.

rsID	Nearest Gene [Orientation]	All Genes in Region ± 50,000 bp
rs423559749	*LOC114114557* (*TRBV6-2*) [exonic]	*LOC121819444* (*TRBV3-1*), *LOC121816042*, *LOC101114438* (*TRBV7-9*), *LOC101114950* (*TRBV5-5*), *LOC114114445* (*TRBV6-2*), *LOC105613885*, *LOC114114557* (*TRBV6-2*), *LOC101115704* (*TRBV5-6*), *LOC121819450* (*TRBV5-1*), *LOC121819446* (*TRBV5-1*), *LOC132657147* (*TRBV6-9*), *LOC114114447* (*TRBV6-2*), *LOC114114448* (*TRBV6-1*), *LOC121819457* (*TRBV5-5*), *LOC132659772*, *LOC114114558* (*TRBV9*)
rs401427029	*TTC39C* [intronic]	*TTC39C*, *LOC114110499*
rs407174590	*ADAR* [5′]	*ADAR*, *KCNN3*
rs418816169	*PIEZO2* [intronic]	*PIEZO2*
rs430733414	*SETBP1* [intronic]	*SETBP1*
rs403221986		
rs427745519		
rs413519109		
rs161045311	*SOX15* [exonic], *EIF4A1* [exonic], *CD68* [exonic]	*LOC114116879*, *TNFSF12*, *TNFSF13*, *SENP3*, *EIF4A1*, *LOC114117084* (*SNORA48*), *LOC114117085* (*SNORA48*), *LOC114117101* (*SNORD10*), *LOC114117063* (*SNORA67*), *SOX15*, *CD68*, *MPDU1*, *FXR2*, *SAT2*, *SHBG*, *ATP1B2*, *TP53*, *WRAP53*, *EFNB3*, *DNAH2*
rs430590929	*SOX15* [exonic], *EIF4A1* [exonic], *CD68* [intronic]	
rs161045330		
rs161045389	*MPDU1* [exonic], *SOX15* [exonic]	
rs401081841	*FXR2* [intronic]	
rs419921875		
rs421850429	*SHBG* [3′]	
rs162128226	*TP53* [intronic]	
rs417779412		
rs399877817	*WRAP53* [intronic]	
rs161046190		
rs426045550	*DNAH2* [intronic]	
rs425193551		
rs161050016	*MFSD6L* [exonic]	*MFSD6L*, *PIK3R6*, *CCDC42*
rs420045693	*NTN1* [intronic]	*NTN1*
rs420358530	*GSG1L2* [exonic]	*USP43*, *DHRS7C*, *GSG1L2*, *GLP2R*

**Table 5:** Summary of ROH that were present in ≥ 70% of a breed within the ROH (1 Mb) and ROH (0.5 Mb) analyses. For each ROH, * indicates the statistics for the breed with ≥ 70% individuals called in the run. The ROH length was calculated as the number of bp between the initial and final SNP positions.

		ROH (0.5 Mb)	ROH (0.5 Mb)	ROH (0.5 Mb)	ROH (1 Mb)	ROH (0.5 Mb)	ROH (1 Mb)	ROH (0.5 Mb)
Chr:Start-End		2:114,908,538–115,694,442	2:123,322,344–123,534,483	3:153,875,227–154,922,651	3:153,959,687–154,922,651	6:37,715,516–39,354,587		10:29,230,492–29,662,445
ROH Length		785,905	212,140	1,047,425	962,965	1,639,072	1,639,072	431,954
SNP Count		98	32	157	141	199	199	61
Polypay	Min %	24.06	31.83	6.77	7.02	33.83	33.83	40.60
	Max %	51.63	40.60	11.53	8.02	41.35	41.35	57.64
Rambouillet	Min %	37.21	70.89 *	10.40	7.69	80.46 *	79.63 *	33.06
	Max %	42.00	71.73 *	18.09	8.52	95.63 *	94.39 *	38.46
Suffolk	Min %	72.11 *	29.75	70.04 *	72.11 *	39.88	31.82	70.66 *
	Max %	79.13 *	30.17	77.48 *	73.76 *	49.59	45.45	72.93 *

**Table 6:** Summary of genes contained within ROH signatures of selection. * indicates the information is relevant for the ROH (0.5 Mb) analysis but not the ROH (1.0 Mb) analysis of the given region.

Region of ROH Island (±50,000 bp)	Breed and ROH Analysis	Genes
2:114,858,538–115,744,442	Suffolk ROH (0.5 Mb)	*FAM168B*, *PLEKHB2*, *LOC105608693*, *LOC114113320*, *LOC101106144* (*EEF1A1*), *LOC101106402* (*ARPC4*), *LOC106990902* (*MSX2*)
2:123,272,344–123,584,483	Rambouillet ROH (0.5 Mb)	*LOC101120641* (*MTIF3*), *LOC101122056* (*TMED2*)
3:153,825,227 */153,909,687–154,972,651	Suffolk ROH (0.5 Mb) and ROH (1 Mb)	LLPH *, *HMGA2*, *LOC132659626*, *LOC121819124*, *MSRB3*, *LOC105609948* (*UBE2D3*), *LEMD3*, *WIF1*, *LOC114114191*
6:37,665,516–39,404,587	Rambouillet ROH (0.5 Mb) ROH (1 Mb)	*TRNAA-CGC*, *LAP3*, *MED28*, *FAM184B*, *DCAF16*, *NCAPG*, *LCORL*, *LOC132660032* (*CFDP2*), *LOC101104580* (*SET*), *LOC121819821*
10:29,180,492–29,712,445	Suffolk ROH (0.5Mb)	*FRY*, *LOC121820437*, *LOC101110773* (*EEF1A1*), *RXFP2*

**Table 7:** Results of F_ST_ analyses. Eighteen SNPs were significant by both Rambouillet–Suffolk (R-S) and Rambouillet–Polypay (R-P) and insignificant by Polypay–Suffolk (P-S) F_ST_ analyses. Genes within ± 50,000 bp of each SNP were proposed as candidate genes.

rsID	Chr	Pos	R-S F_ST_	R-P F_ST_	P-S F_ST_	Genes within ± 50,000 bp
rs421362086	1	226,945,393	0.484	0.399	0.016	*LOC114113867*, *LOC101104288*
rs424868844	2	214,749,839	0.560	0.410	0.050	--
rs430105435	2	214,757,669	0.560	0.412	0.050	--
rs422033355	3	122,187,178	0.480	0.364	0.027	*LOC121819172*
rs159911297	3	211,616,229	0.532	0.331	0.071	*TIGAR*, *CCND2*, *LOC105608579*, *LOC121819220*, *LOC132659637*
rs408105992	4	105,852,382	0.471	0.393	0.011	*TMEM178B*
rs423710982	6	37,132,187	0.486	0.367	0.037	*PPM1K*, *ABCG2*
rs403906365	8	61,597,763	0.488	0.325	0.059	*BCLAF1*, *LOC105608893*
rs418992975	10	73,712,633	0.490	0.409	0.013	*LOC132657267*, *MBNL2*
rs401322014	14	43,365,324	0.474	0.369	0.024	*PEPD*, *LOC132657685*
rs429950267	14	43,367,902	0.474	0.369	0.024	*PEPD*, *LOC132657685*
rs406252998	16	2,861,069	0.492	0.364	0.026	*KCNIP1*, *GABRP*
rs417106434	16	20,059,879	0.471	0.337	0.031	*PDE4D*, *TRNAH-AUG_7*
rs426980318	18	29,393,657	0.497	0.352	0.051	*ETFA*, *LOC105603147*, *ISL2*, *LOC132658148*, *SCAPER*
rs401153126	18	33,166,790	0.499	0.404	0.016	--
rs413553624	20	30,923,860	0.472	0.387	0.012	*LOC101113632*, *LOC101113108*, *LOC114109686*, *LOC101113369*, *LOC101112606*, *H1-1*, *TRIM38*, *LOC132658266*, *SLC17A2*, *SLC17A3*
rs400967878	23	44,688,779	0.481	0.331	0.041	*SETBP1*
rs402778861	23	44,691,416	0.488	0.332	0.043	*SETBP1*

**Table 8:** Literature context for genes implicated by GWAS for LFW, AFW and PLEW. These genes have been previously identified in signature of selection, DEG analyses and transcriptomics characterizations of hair follicles in sheep or other species.

Gene	Trait	Previous Analyses	Reference
*ADAR*	AFW	DEG for hair follicle development in Merino sheep; transcriptomics analysis of sheep skin and other species	[40,41]
*ATP1B2*	PLEW	DEG for hair follicle development in Merino sheep	[40]
*CD68*	PLEW	DEG in Yak hair cycle	[42]
*DHRS7C*	PLEW	DEG in Yak hair cycle	[42]
*EFNB3*	PLEW	DEG for hair follicle development in Merino sheep; signature of selection analysis for domestication	[40,43]
*EIF4A1*	PLEW	Expressed in Suzhou sheep skin	[44]
*GLP2R*	PLEW	DEG for hair follicle development in Merino sheep	[40]
*KCNN3*	AFW	DEG for hair follicle development in Merino sheep; transcriptomics analysis of sheep skin and other species; DEG in Yak hair cycle; DEG between anagen and telogen phases in Pashmina fiber	[40,41,42,45]
*LOC101114438* (*TRBV7-9*)	LFW	Signature of selection analysis for wool fineness	[43]
*NTN1*	PLEW	DEG between anagen and telogen phases in Pashmina fiber; DEG for hair follicle development in Merino sheep; DEG in coarse vs. fine wool; signature of selection analysis for wool fineness; transcriptomics analysis of sheep skin and other species	[40,41,43,45,46]
*PIEZO2*	AFW	DEG in Yak hair cycle	[42]
*PIK3R6*	PLEW	Transcriptomics analysis of sheep skin and other species	[41]
*SETBP1*	AFW	DEG in Yak hair cycle	[42]
*SHBG*	PLEW	DEG in Yak hair cycle	[42]
*SOX15*	PLEW	DEG for hair follicle density in Hetian sheep	[47]
*TNFSF12*	PLEW	DEG between anagen and telogen phases in Pashmina fiber; expressed in Suzhou sheep skin; DEG in Yak hair cycle	[42,44,45]
*TNFSF13*	PLEW	DEG in Yak hair cycle	[42]
*TP53*	PLEW	Expressed in Suzhou sheep skin	[44]
*WRAP53*	PLEW	Signature of selection analysis for domestication	[43]

## Data Availability

Data is contained within the article or Supplementary Material.

## Supplementary Materials

The following supporting information can be downloaded at: https://www.mdpi.com/article/10.3390/genes16070733/s1, Supplementary File S1, Figure S1: PCA with the USSES GWAS dataset. The proportion of variance explained by each PC is given in parentheses. Supplementary File S1, Figure S2: Pearson correlation coefficients between LFW, AFW, PLEW and longevity within breeds. All correlations were significant with *p*-values < 0.05. (A) Correlations within Columbia sheep. (B) Correlations within Polypay sheep. (C) Correlations within Rambouillet sheep. (D) Correlations within Suffolk sheep. In all panels, correlation coefficients below 0 are denoted by the red scale, and correlation coefficients above 0 are denoted by the blue scale, with darker colors being closest to ±1. Supplementary File S1, Figure S3: Distribution of inbreeding coefficients from the method-of-moments inbreeding (FMOM) and ROH-based inbreeding (FROH) calculations. The horizontal red line represents the mean inbreeding coefficient. Supplementary File S2, Table S1: Results of Tukey HSD multiple comparison of means analysis. The mean difference and adjust *p*-value for each pairwise breed comparison are provided. Supplementary File S2, Table S2: Pearson correlation *p*-values for across- and within-breed correlation analyses. Supplementary File S2, Table S3: Descriptive statistics of inbreeding coefficients from the inbreeding and homozygosity analyses calculated from the signature of selection dataset (USSES and Repository sheep). The breed average, standard deviation, minimum value and maximum value are given for the method-of-moments inbreeding coefficient (FMOM), ROH-based inbreeding coefficient (FROH), observed homozygosity and expected homozygosity. Supplementary File S2, Table S4: Tukey HSD *p*-value results for inbreeding analyses. Supplementary File S3, Sheet 1 Genes of Interest: List of genes of interest identified through GWAS, ROH and F_ST_ analyses. Supplementary File S3, Sheet 2 Descriptive Stats by Genotype: Descriptive statistics (mean and standard deviation) of LFW, AFW and PLEW traits by GWAS SNP genotype. Supplementary File S3, Sheet 3 LD (r2) GWAS SNPs: The LD estimations (*r^2^*) for GWAS SNPs on chromosomes 11 and 23. The LD estimation for each pair of SNPs and haplotype frequencies are reported. Supplementary File S3, Sheet 4 ROH Frequency by Genotype: Genotypic frequencies by breed for SNPs contained within ROH. Supplementary File S3, Sheet 5 F_ST_ SNP Frequencies: Genotypic frequencies by breed for SNPs identified through F_ST_ analysis. Supplementary File S3, Sheet 6 F_ST_ *p*-values: Estimated F_ST_ and *p*-value statistics for SNPs reported in F_ST_ analysis. Supplementary File S3, Sheet 7 Significant Genotypes: Phenotype and genotype information for all samples and significant SNPs identified in the current study. Supplementary File S3, Sheet 8 LFW AFW GWAS Iterations: Comparison of *p*-values for both versions (with and without PLEW covariate) of LFW and AFW GWAS. Supplementary File S4: Boxplot figures showing the distribution of LFW, AFW and PLEW by breed and genotype for each genome-wide significant SNP.

## Abbreviations

The following abbreviations are used in this manuscript:

LFW	Lifetime fleece weight (kg)
AFW	Average fleece weight (kg)
PLEW	Average post-lambing ewe weight (kg)
