# Supplementary material for: Genome-Wide Associations with Body and Fleece Weight in United States Sheep
Source: Genes (Basel). 2025 Jun 24;16(7):733. doi: 10.3390/genes16070733 (PMC12294545; doi:10.3390/genes16070733)
Supplement: Supplementary file 1 [file genes-16-00733-s001.zip › Additional_File_S1.pdf]

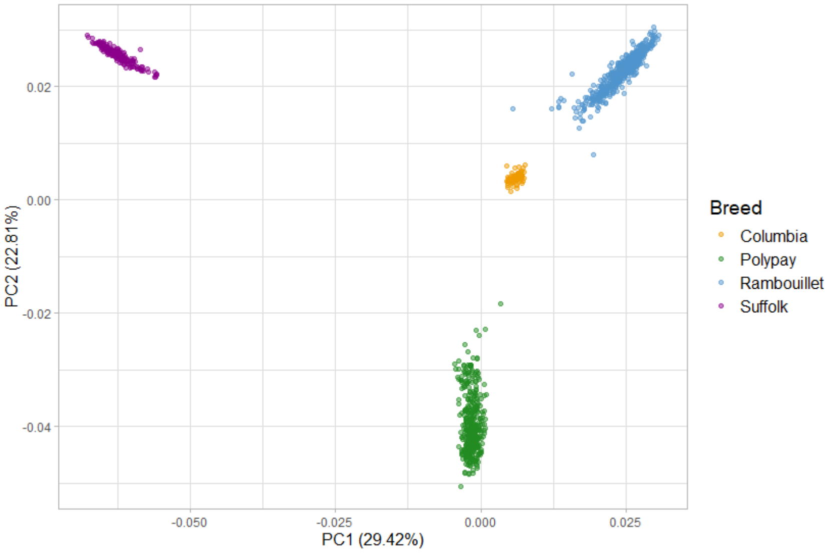

Supplementary Figure S1. PCA with the USSES GWAS dataset. The proportion of variance explained by each PC are given in parentheses.

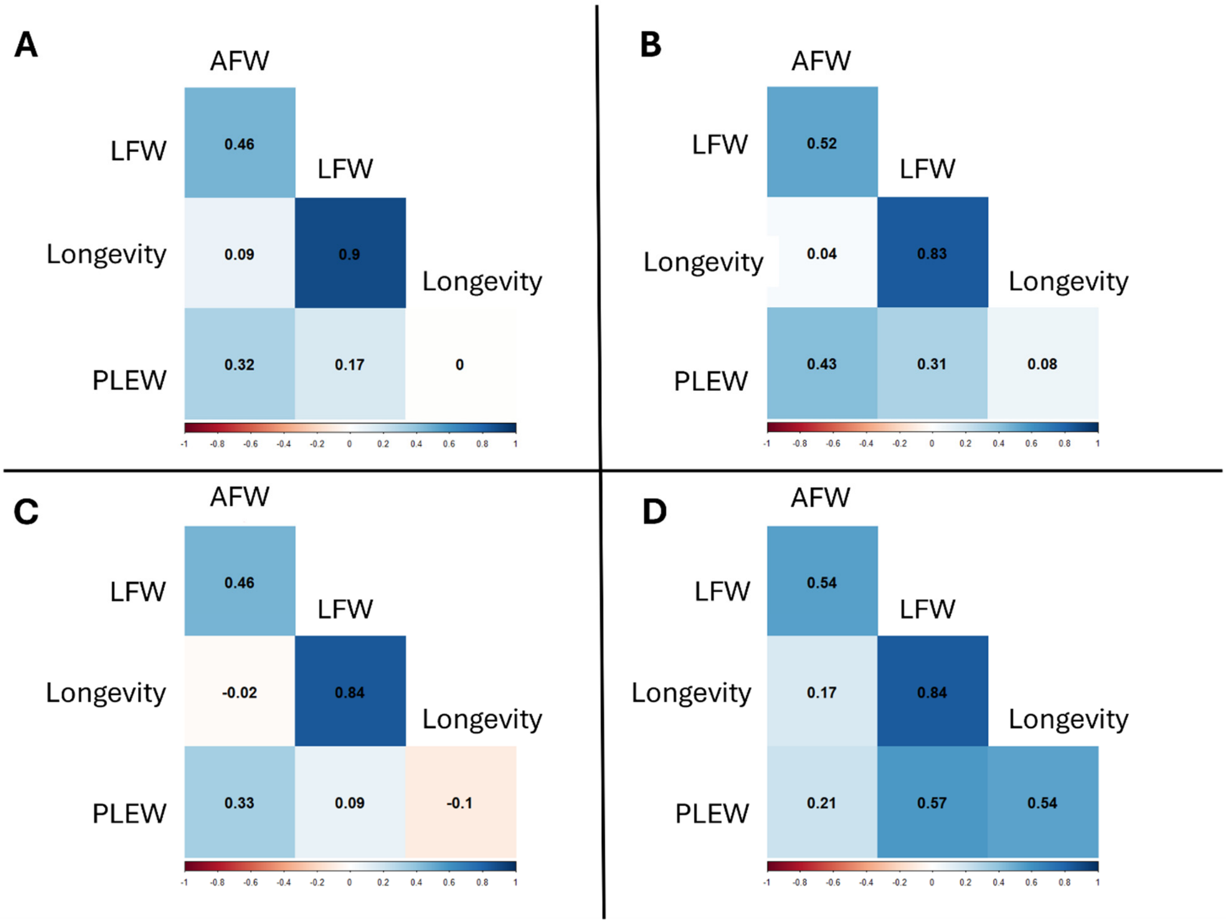

Supplementary Figure S2. Pearson correlation coefficients between LFW, AFW, PLEW and longevity within breeds. All correlations were significant with p-values < 0.05. (A) Correlations within Columbia sheep (B) Correlations within Polypay sheep (C) Correlations within Rambouillet sheep (D) Correlations within Suffolk sheep. In all panels, correlation coefficients below 0 are denoted by the red scale and correlation coefficients above 0 are denoted by the blue scale, with darker colors being closest to ±1.

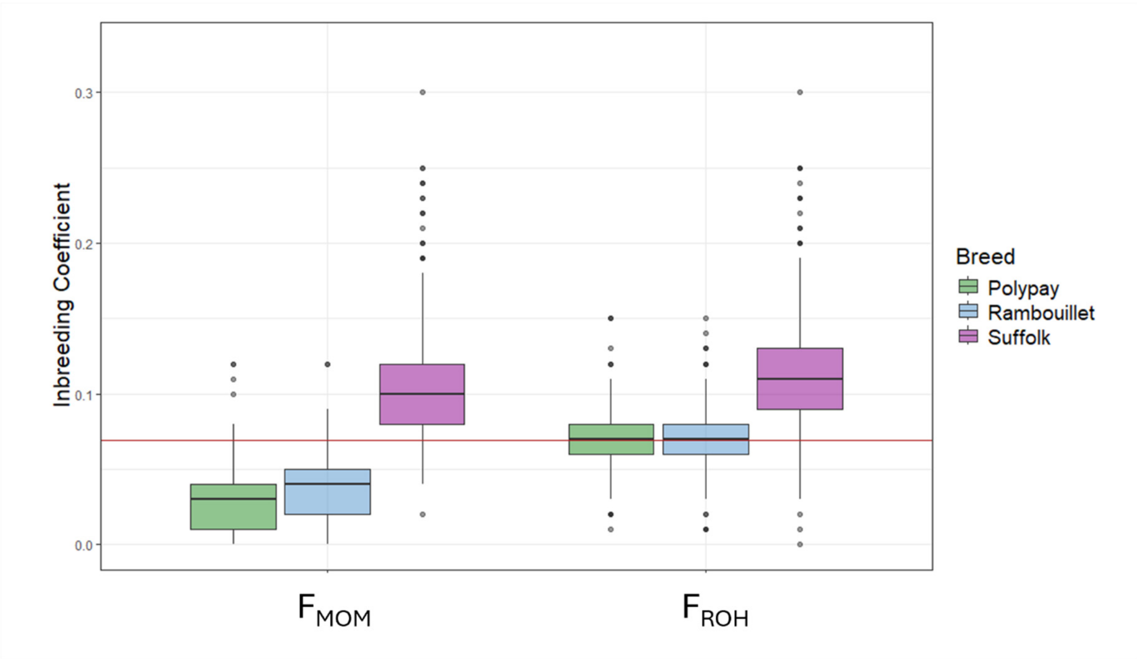

Supplementary Figure S3. Distribution of inbreeding coefficients from the method-of-moments inbreeding (F_{MOM}) and ROH-based inbreeding (F_{ROH}) calculations. The horizontal red line represents the mean inbreeding coefficient.
