# Supplementary material for: Genome-Wide Associations with Body and Fleece Weight in United States Sheep
Source: Genes (Basel). 2025 Jun 24;16(7):733. doi: 10.3390/genes16070733 (PMC12294545; doi:10.3390/genes16070733)
Supplement: Supplementary file 1 [file genes-16-00733-s001.zip › Additional_File_S4.pdf]

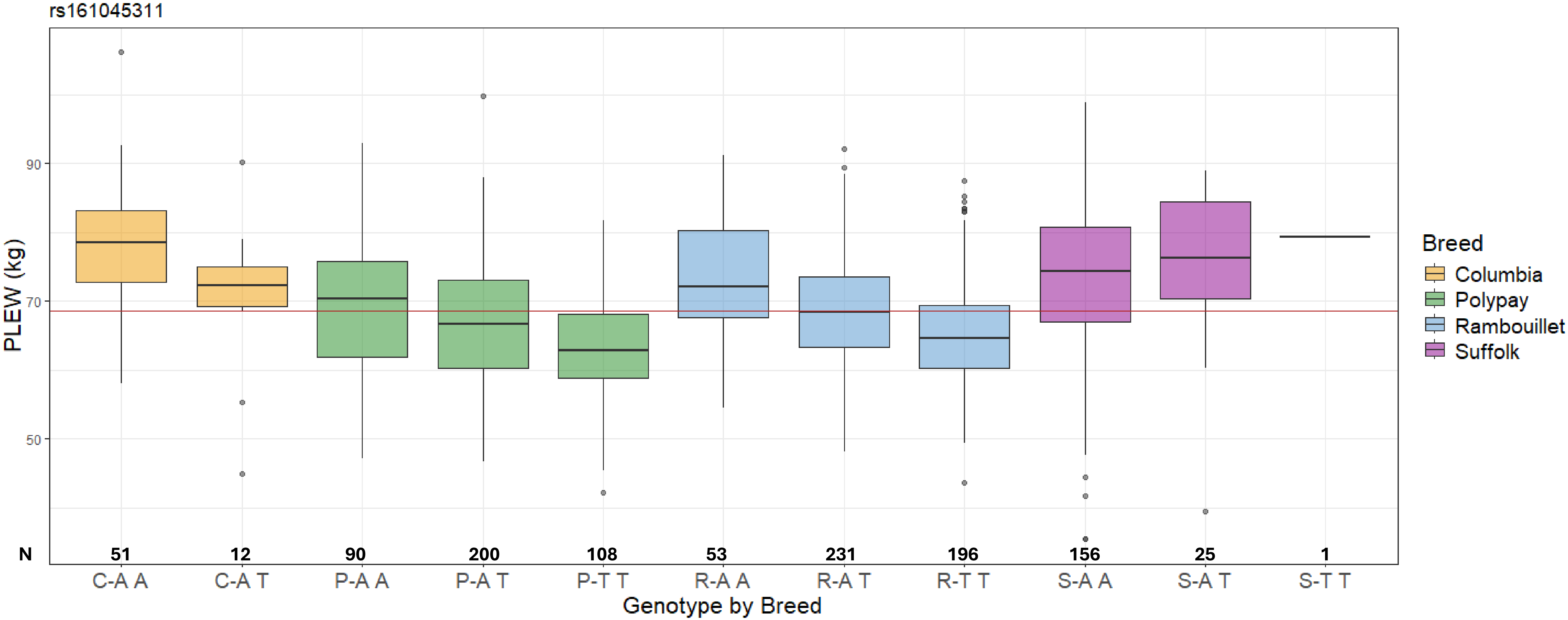

Additional File S4. Distribution of PLEW by rs161045311 genotype. The horizontal line represents the trait average.

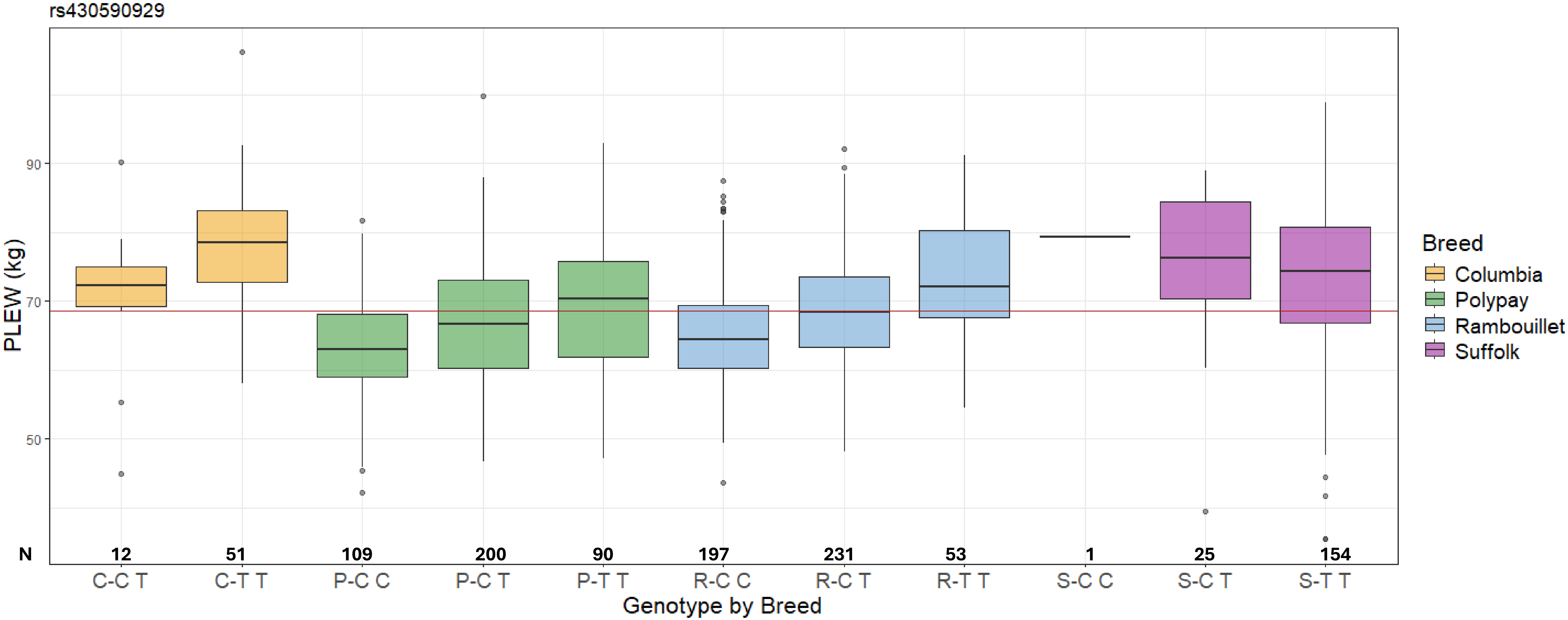

Additional File 4, Figure 2. Distribution of PLEW by rs430590929 genotype. The horizontal line represents the trait average.

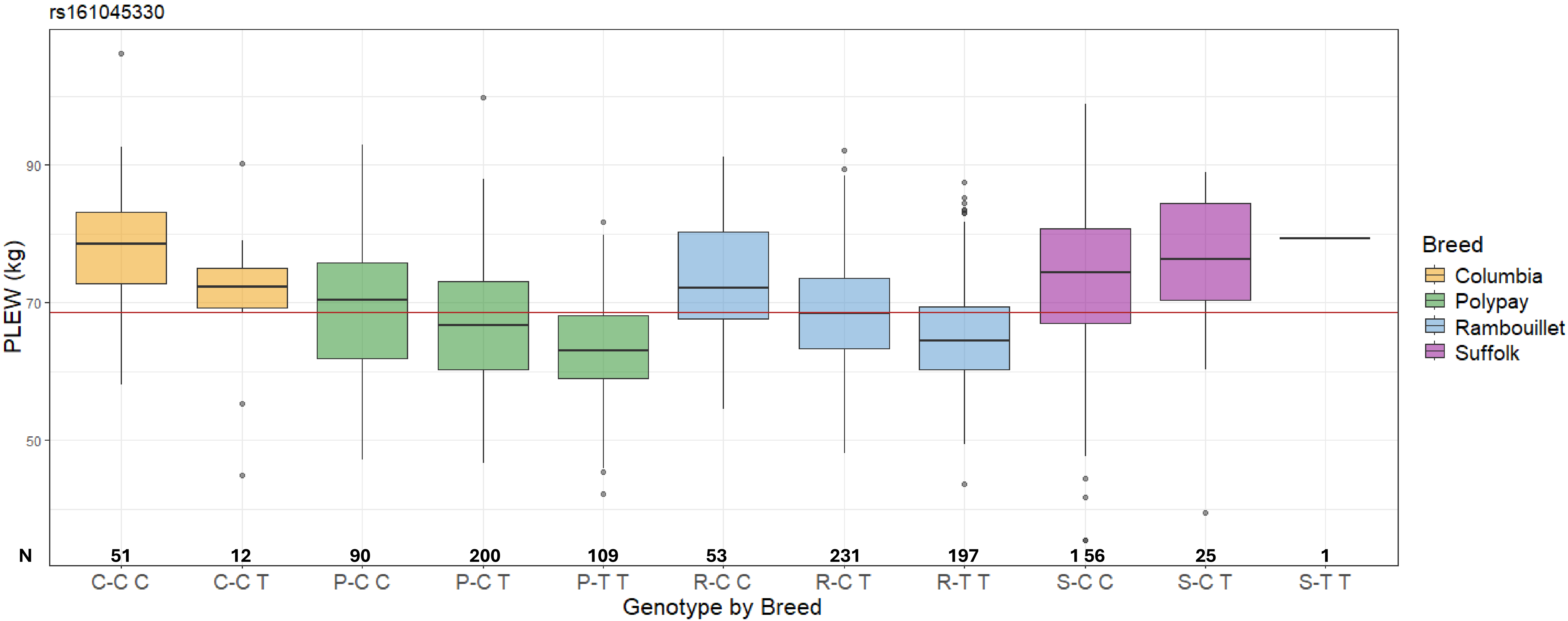

Additional File 4, Figure 3. Distribution of PLEW by rs161045330 genotype. The horizontal line represents the trait average.

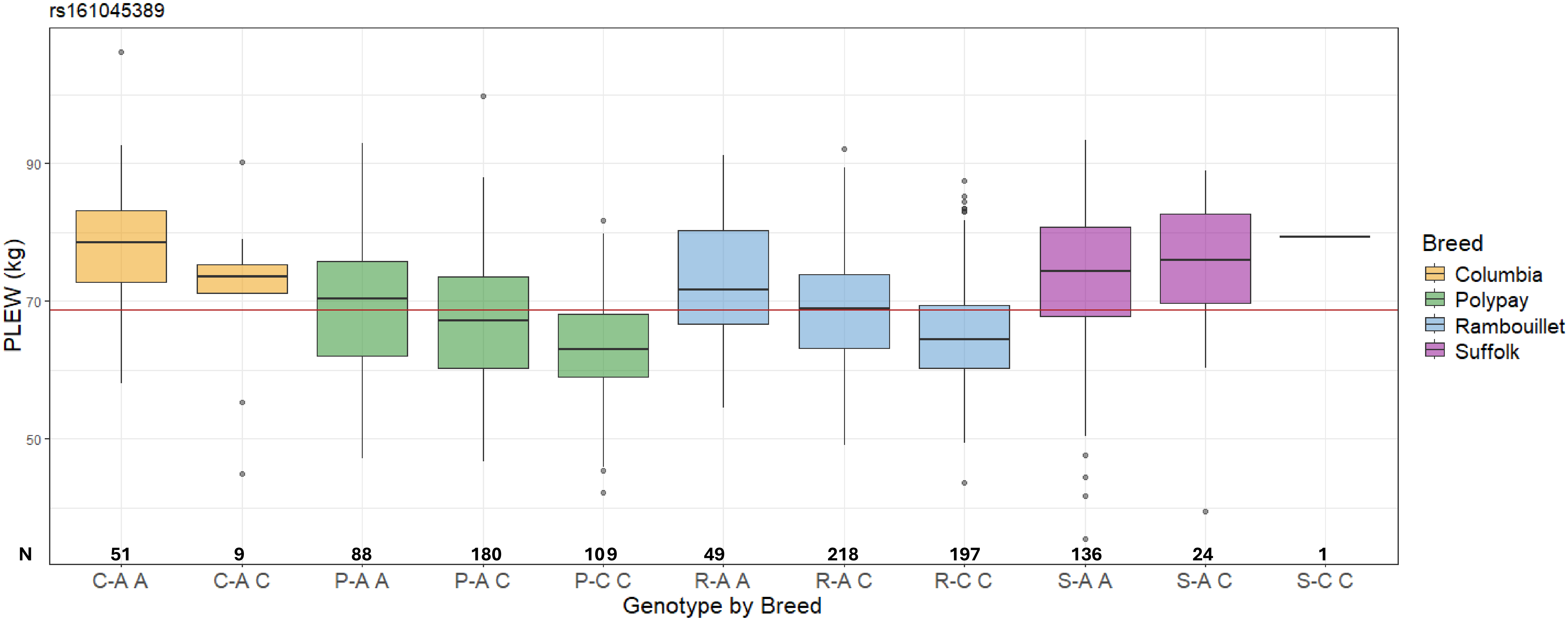

Additional File 4, Figure 4. Distribution of PLEW by rs161045389 genotype. The horizontal line represents the trait average.

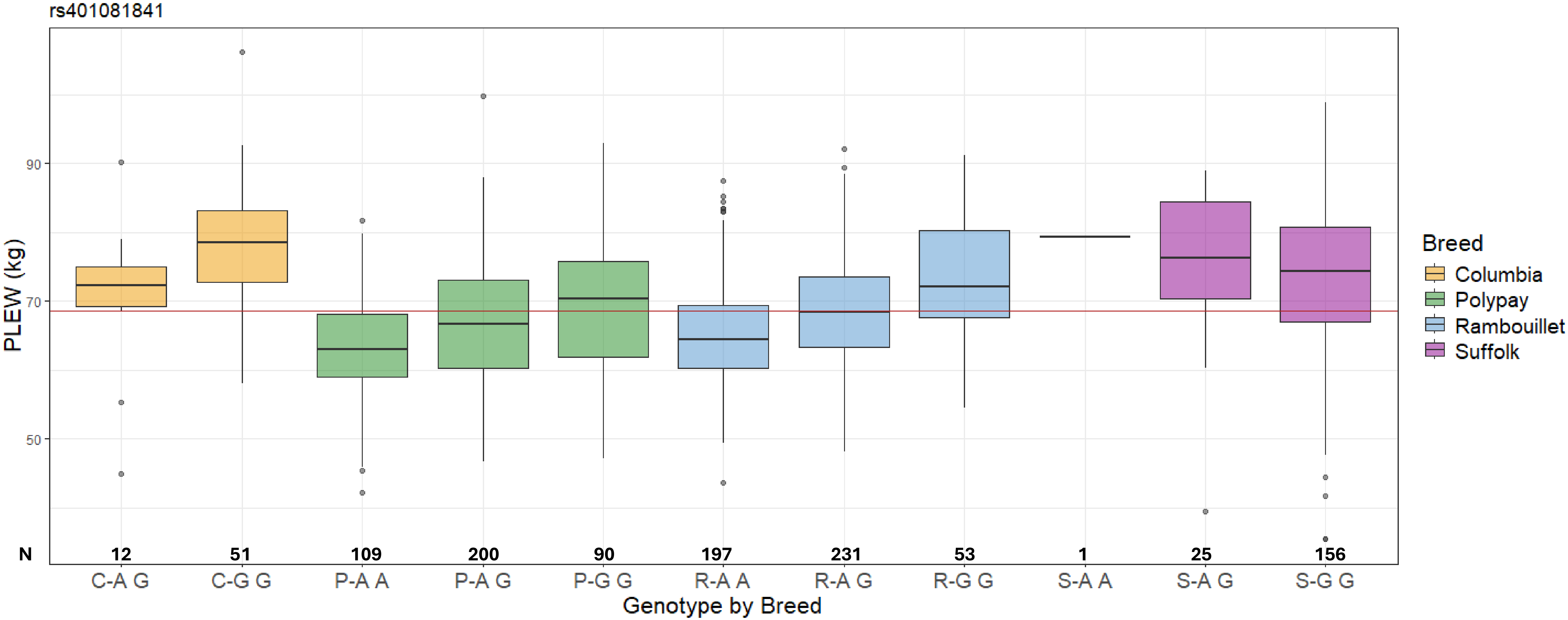

Additional File 4, Figure 5. Distribution of PLEW by rs401081841 genotype. The horizontal line represents the trait average.

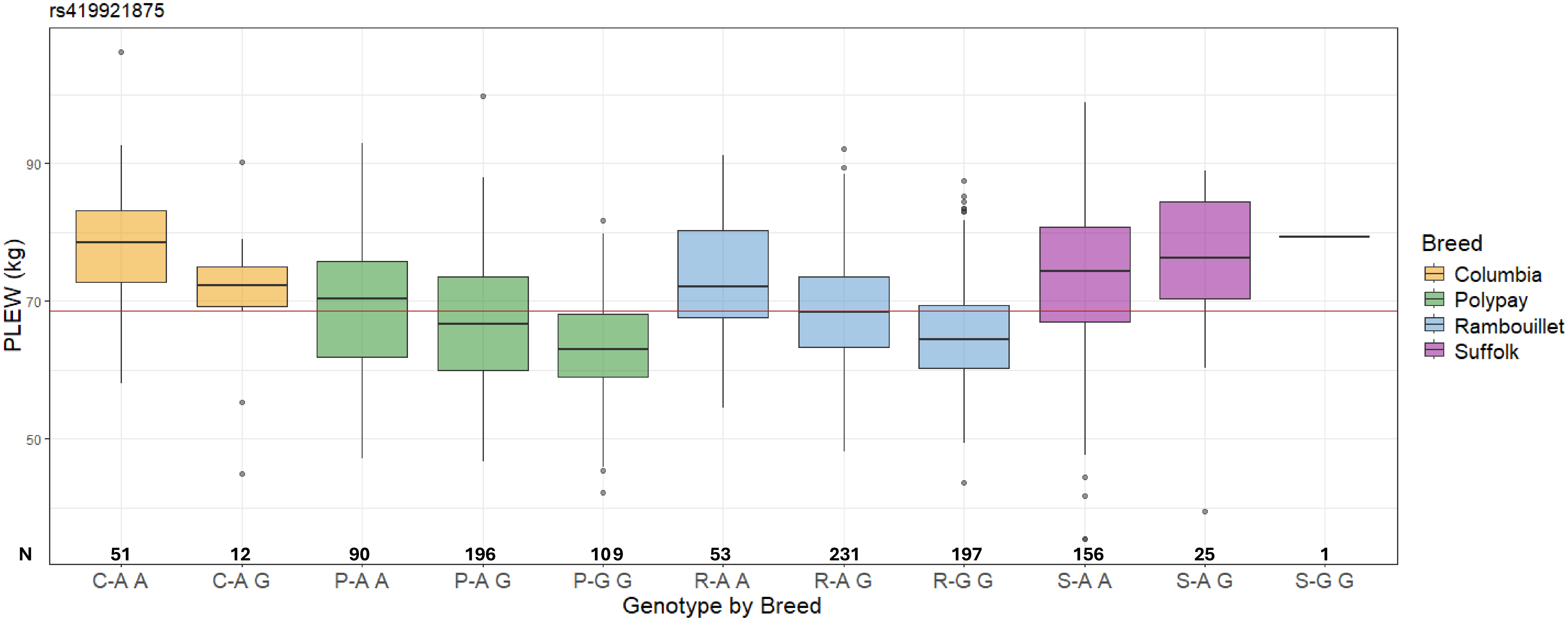

Additional File 4, Figure 6. Distribution of PLEW by rs419921875 genotype. The horizontal line represents the trait average.

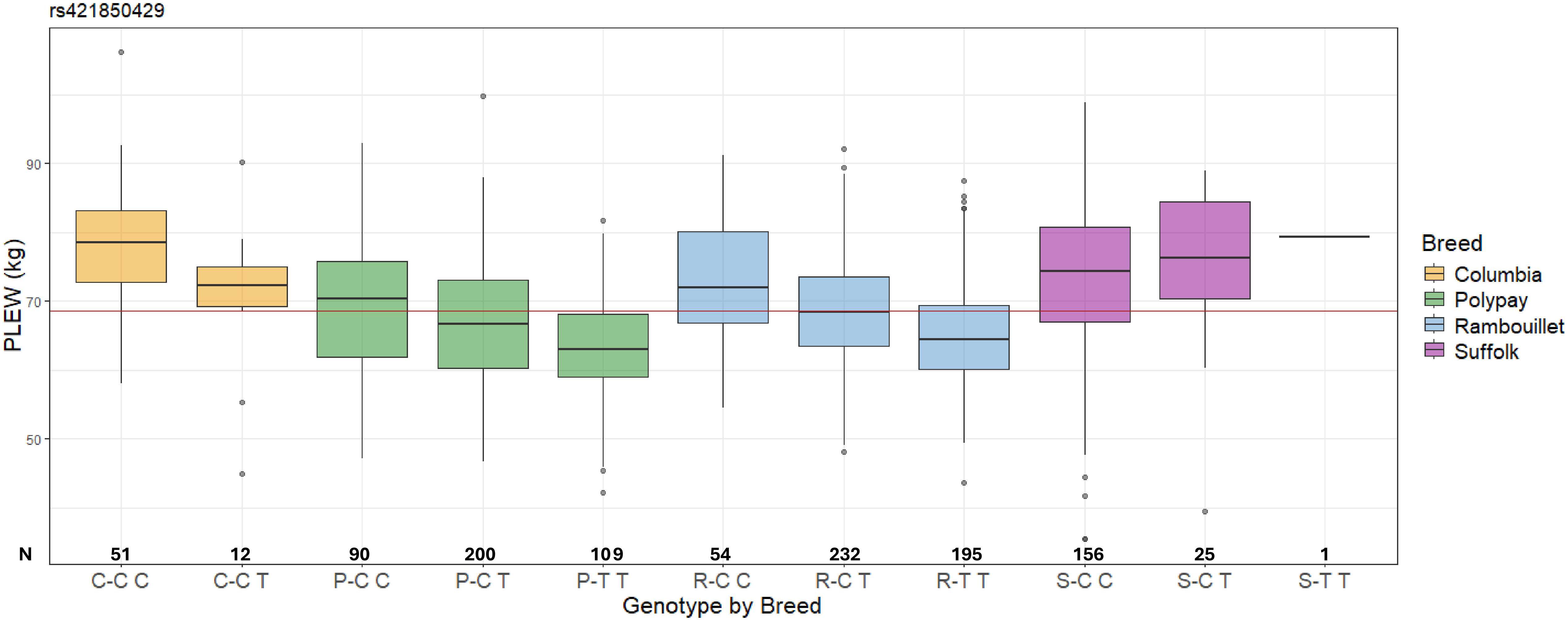

Additional File 4, Figure 7. Distribution of PLEW by rs421850429 genotype. The horizontal line represents the trait average.

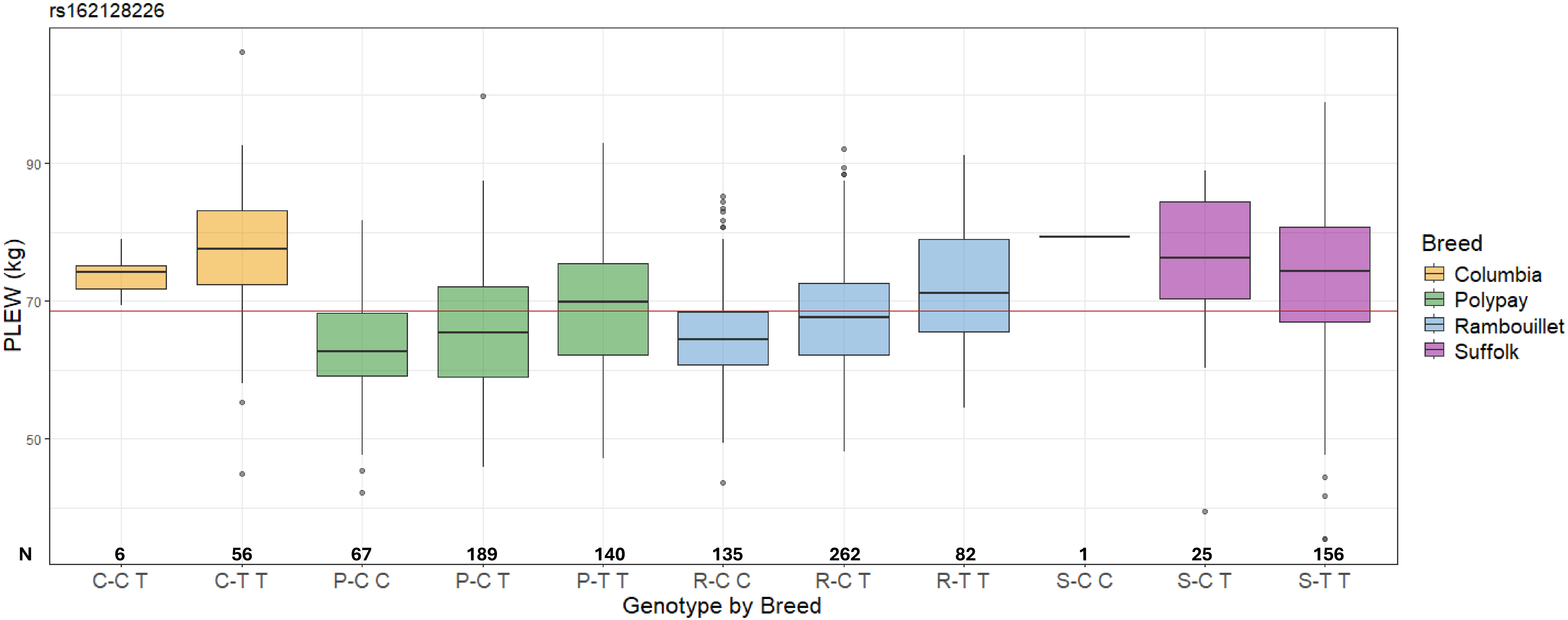

Additional File 4, Figure 8. Distribution of PLEW by rs162128226 genotype. The horizontal line represents the trait average.

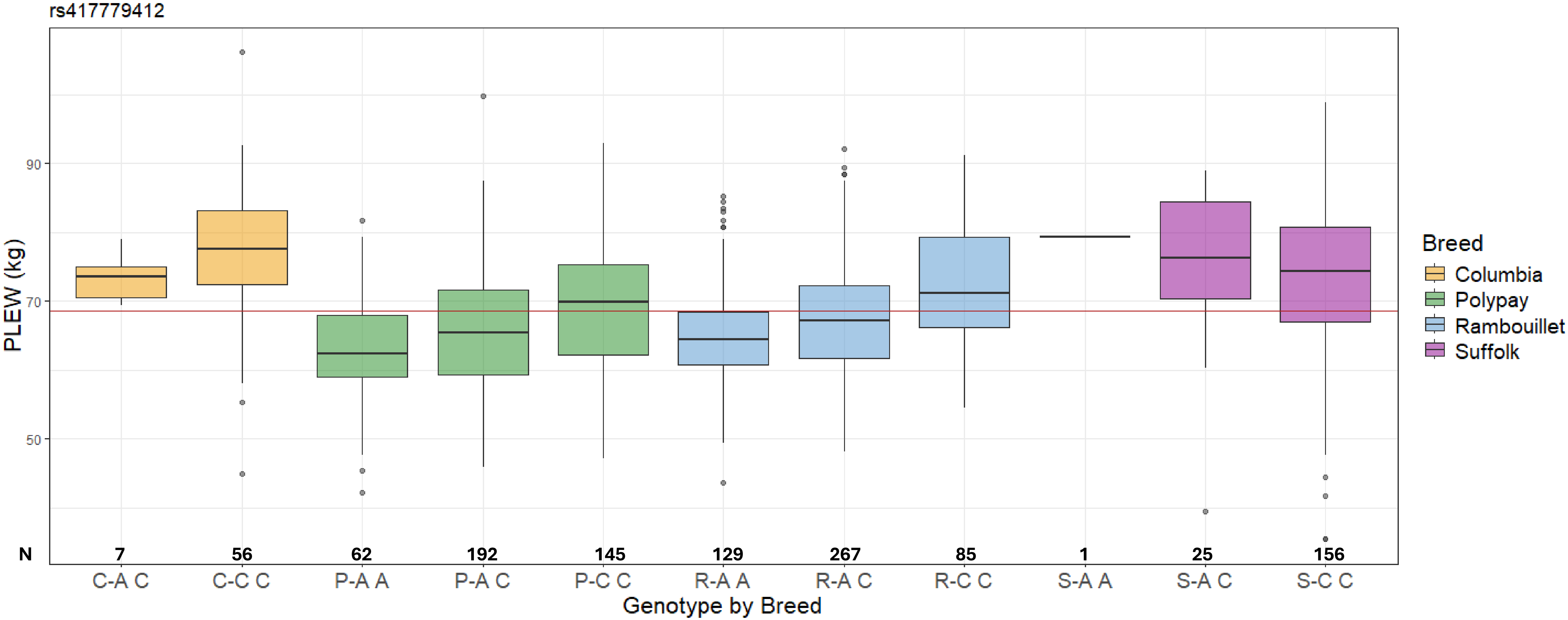

Additional File 4, Figure 9. Distribution of PLEW by rs417779412 genotype. The horizontal line represents the trait average.

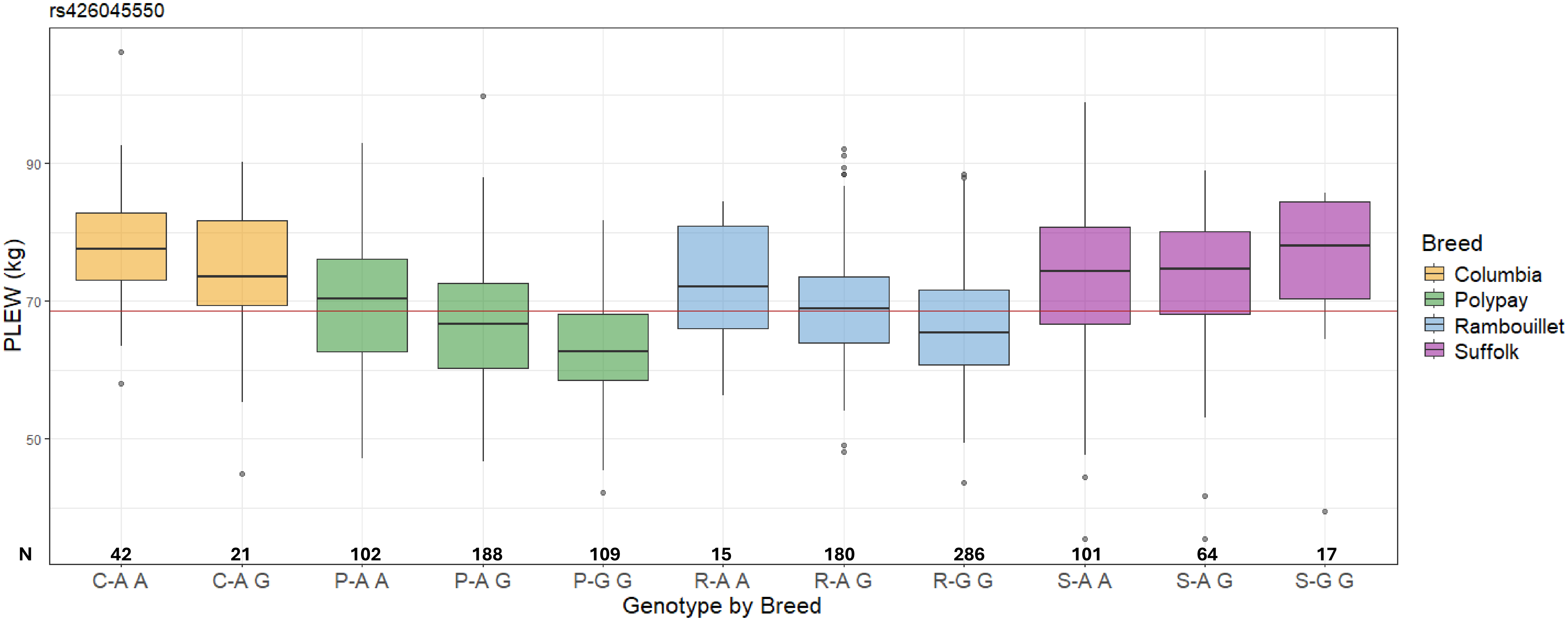

Additional File 4, Figure 10. Distribution of PLEW by rs426045550 genotype. The horizontal line represents the trait average.

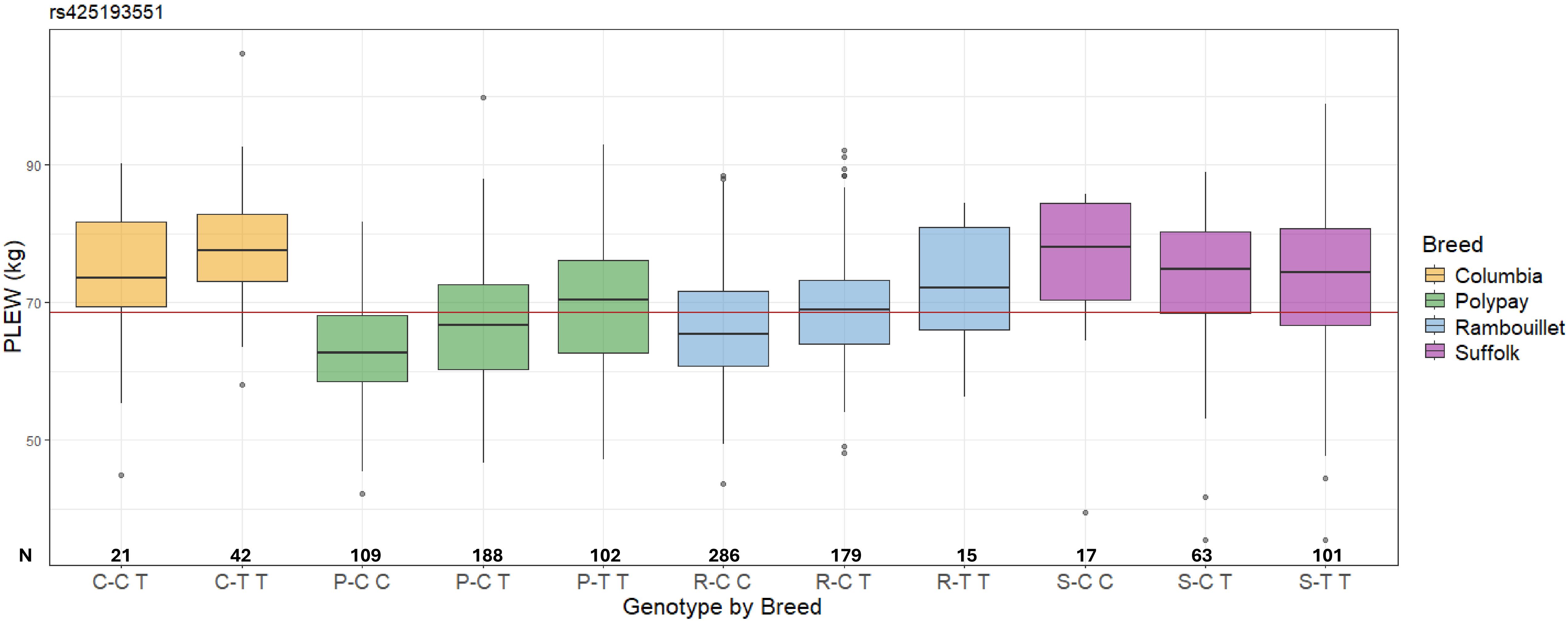

Additional File 4, Figure 11. Distribution of PLEW by rs425193551 genotype. The horizontal line represents the trait average.

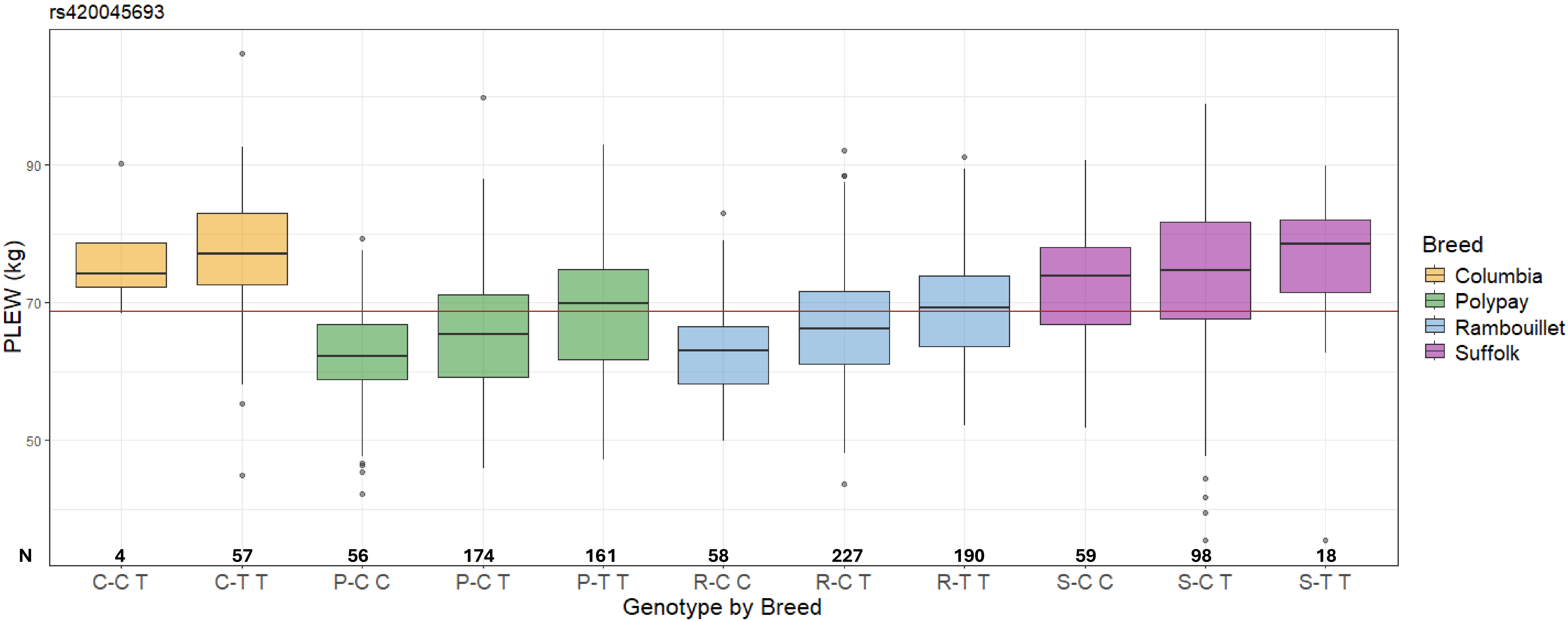

Additional File 4, Figure 12. Distribution of PLEW by rs420045693 genotype. The horizontal line represents the trait average.

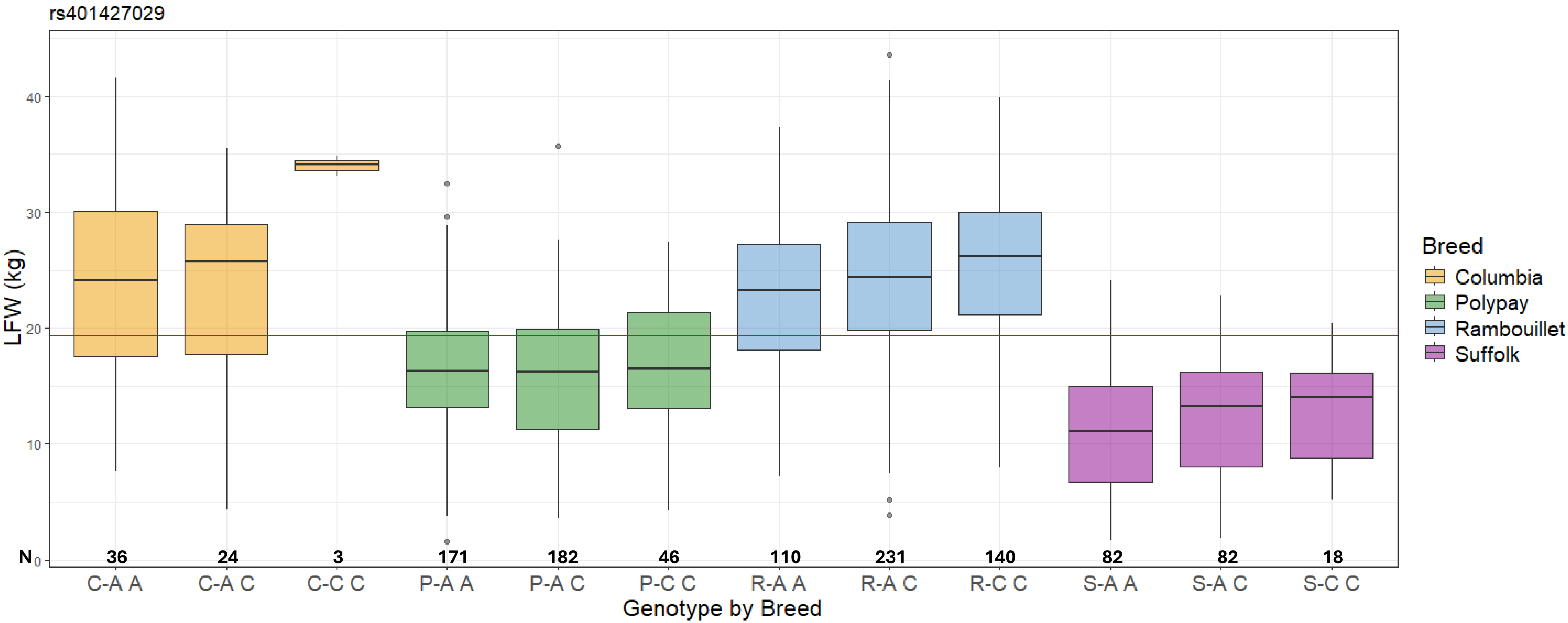

Additional File 4, Figure 13. Distribution of LFW by rs401427029 genotype. The horizontal line represents the trait average.

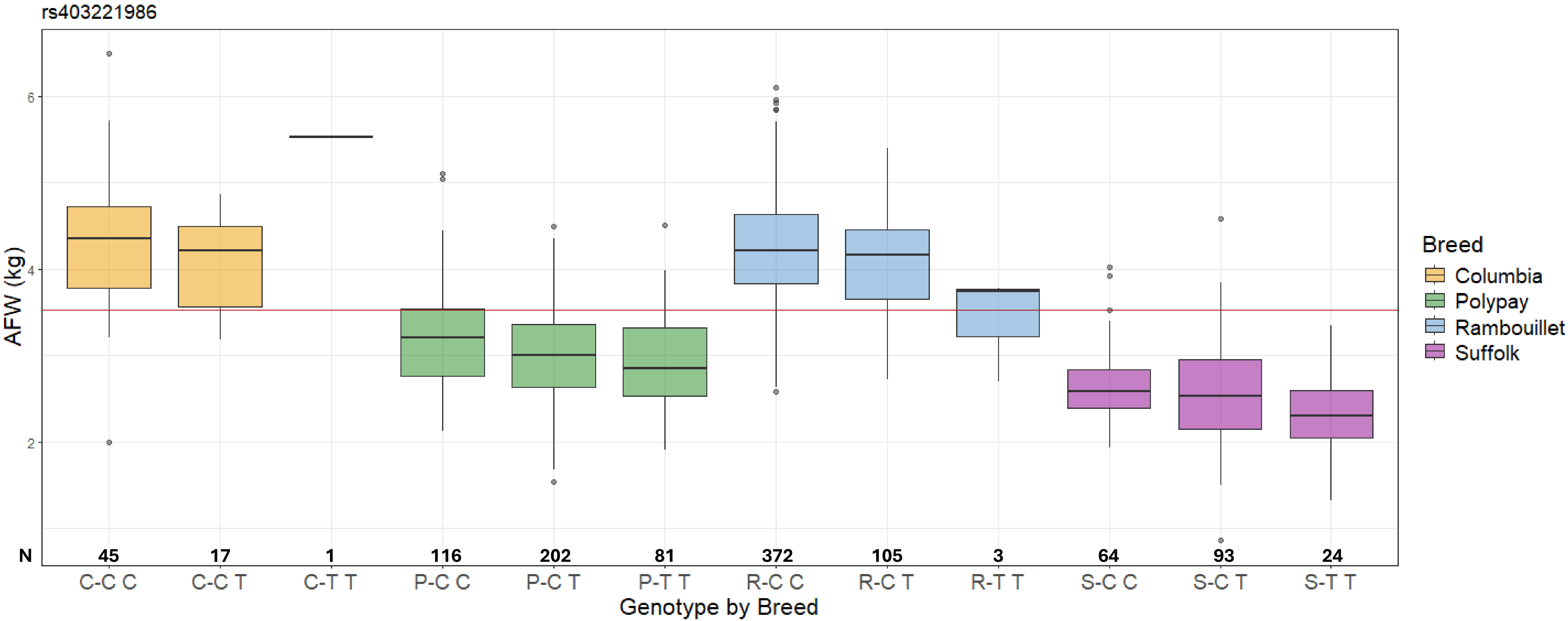

Additional File 4, Figure 14. Distribution of AFW by rs403221986 genotype. The horizontal line represents the trait average.

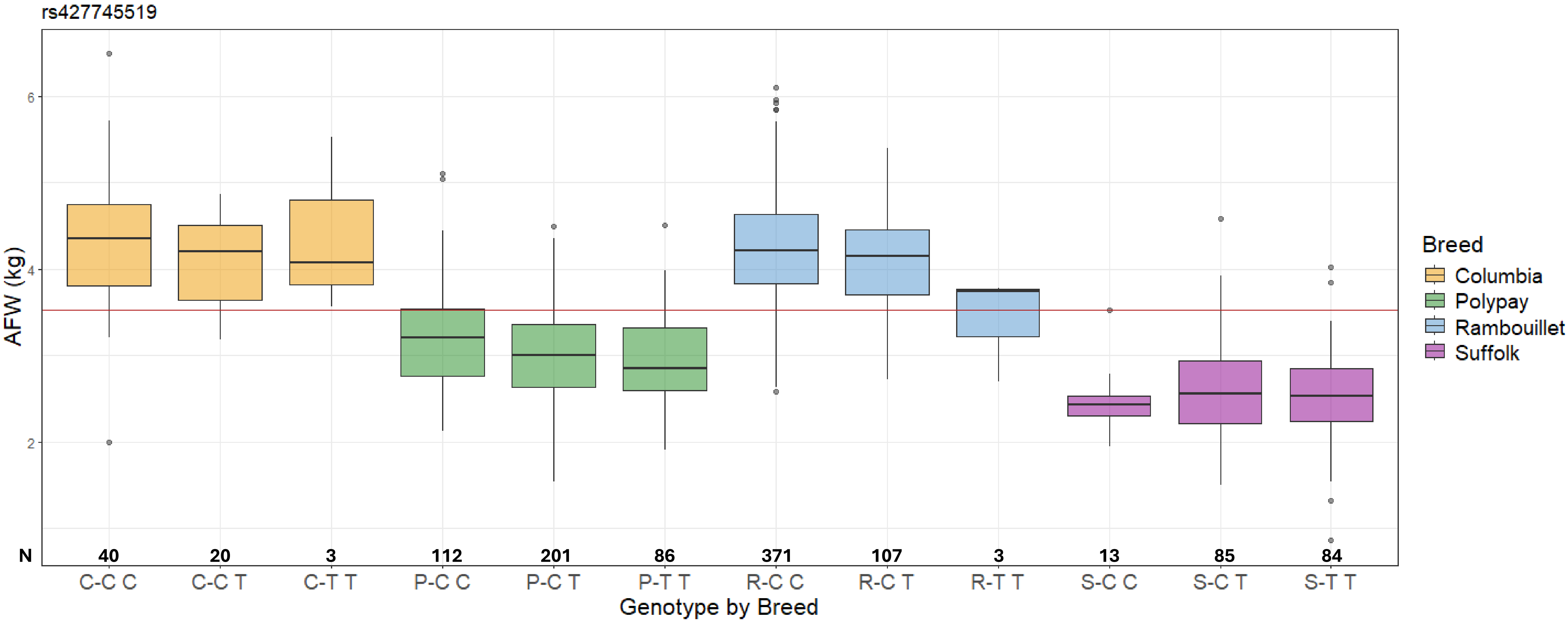

Additional File 4, Figure 15. Distribution of AFW by rs427745519 genotype. The horizontal line represents the trait average.
